# Probabilistic brain MR image transformation using generative models

**DOI:** 10.1038/s41598-025-31958-3

**Published:** 2025-12-20

**Authors:** Sepideh Rezvani, Saeed Moazami, Christina J. Azevedo, Assad A. Oberai

**Affiliations:** 1 Neurology Department, University of Southern California, Los Angeles, 90033 USA; 2 Aerospace and Mechanical Engineering, University of Southern California, Los Angeles, 90089 USA

**Keywords:** Biomedical engineering, Neurology

## Abstract

Brain MR image transformation, which is the process of transforming MR images of one type to another, is critical to several downstream neuroimaging tasks that include brain tissue and lesion volume estimation and lesion detection. In recent years, several deep learning-based methods have been applied to address this task; however, for the most part, they have tended to be deterministic. These methods provide a single transformed output for a given input image with no accompanying measure of confidence in the transformation process. In contrast to this, in this study, we demonstrate how a class of probabilistic conditional generative algorithms can be applied to MR image transformation and quantify the performance of these algorithms. We also demonstrate that the ability to generate multiple transformed images for a given input image can be used to estimate the uncertainty in the output and to detect out-of-distribution (OOD) input images. In particular, we apply conditional Generative Adversarial Networks (cGAN), Noise Conditional Score Networks (NCSN), and Denoising Diffusion Probabilistic Models (DDPM) to transform T1, T2, FLAIR, and proton density (PD) MR images. Through extensive computational experiments, we conclude that the probabilistic algorithms are more accurate than other benchmark methods, and among these, the diffusion models yield the most accurate transformation results. Within the diffusion models, DDPM demonstrates higher performance in terms of similarity metrics, and NCSN exhibits accurate distributional measures and computationally favorable characteristics. We also demonstrate how the generative models can be used to assess the confidence in a given transformation and to detect input images that contain pathology and/or artifacts.

## Introduction

Brain MR imaging is the primary method for diagnosing and monitoring patients with many neurological diseases and for performing brain-related research. Additionally, by tailoring MRI sequences, a variety of image types with different anatomical and pathological details can be acquired. However, in many instances, due to reasons that include the physical condition of a subject, the cost of additional acquisition time, and corruption of data, not all of the desired MR image types can be acquired. This can lead to undesired costs and burdens associated with additional scans and the exclusion of some subjects from a research study. This motivates the need to develop algorithms that can transform one MR image type into another.

Over the years, a wide range of methods have been developed for medical image transformation. More recently, Deep Learning (DL)-based methods have proven to be successful in this field^1,2^. This success stems from the inherent ability of these methods to represent the complex and highly nonlinear mappings that are found in many image transformation tasks. The reader is referred to survey articles on this topic^3,4^.

Medical image transformation can be categorized as inter-modality, which involves the transformation of images of one modality to another, or intra-modality, which involves the transformation from one image type or contrast to another one within the same modality. Examples of the former include transformation between MR, computed tomography (CT), and positron emission tomography images (PET) images^5–8^. Whereas examples of the latter include the transformation of MR images of one type to another. For example, the transformation of T2, T1, and FLAIR MR images. The focus of this work is intra-modality transformation of MR images, and in particular, on the conversion of structural MR images of one type to another.

There are two broad perspectives of viewing the image transformation problem. The first perspective, which is the one that is commonly used, is deterministic. In this case, the transformation itself is deterministic, and for one input, it generates a single instance of the output. Direct inference supervised methods^9^ can be considered the most straightforward DL-based algorithms based on a deterministic formulation. In these methods, the model receives the input image and transforms it using a DL-based network. The network is trained using a loss function that measures the difference between the predicted and the target image. Different choices of network architectures and loss functions yield different methods. For example, Dinkla et al.^10^ use a convolutional neural network (CNN) based encoder-decoder and the mean absolute error loss function to transform brain T1 MR images to CT images. Similarly, Xiang et al.^11^ use a network with multiple embedding blocks and a least squares error loss to transform MR images to CT images.

An alternative perspective of the image transformation problem is the probabilistic approach, which is the main focus of this work. Within this approach, the transformation that maps an input image to the output is assumed to be probabilistic. Therefore, for a single input, it yields multiple possible transformed images that are sampled from a probability distribution conditioned on the input image. A simple thought experiment illustrates why for the brain MR image transformation problem this perspective is more appropriate. It is well known that due to the underlying physics, MR images can display significant variability^12^. Consider an example in which multiple devices are used to capture T2 images for a single subject. All these images would differ from each other and will still be considered valid T2 images for that subject. In this scenario, if we develop a method to convert a T1 image for this subject into a T2 image, then it is clear that the appropriate output is not a single image; rather, it is a collection of images, all of which may be considered as valid T2 images. In other words, probabilistic models aim to capture the conditional distribution of plausible target outputs given an input image. Rather than being manually specified, this distribution is implicitly learned from the training data and reflects the inherent variability present in real MR scans, arising from factors such as subject positioning, scanner and acquisition protocols, anatomical and pathological differences, as well as variations in image quality and noise characteristics.

Generative models are DL-based models that are based on probabilistic principles and are, therefore, appropriate for a broad range of applications^13^, including medical image transformation. Among these, conditional generative adversarial networks (cGANs)^14–16^ have been successfully employed in image-to-image transformation tasks, including MR image transformation^17,18^. More recent studies have extended these approaches using hybrid adversarial architectures, such as MTSR-MRI^19^, which jointly performs modality translation and super-resolution. In general, GAN-based methods are difficult to work with since their training requires working with an adversarial loss, which leads to a min-max optimization problem that is harder to solve than the more common minimization problem. This difficulty is circumvented by diffusion models that generate multiple samples by utilizing the underlying score function of the probability density function and learn this score function by solving a minimization problem^20–22^. In recent years, diffusion models have found a wide range of applications in medical image transformation^23–25^, including the transformation of MR images^26–29^. One drawback of diffusion models is that they incur larger computational costs during the generation process, which happens over several hundred steps. Recently, several studies have focused on reducing computational time while aiming to preserve image quality, often by operating in latent space^27^. For instance, Ozbey et al. combine the diffusion process with an adversarial projection scheme to achieve faster image sampling during inference^30^. Also, Jiang et al. introduce Fast-DDPM, a denoising diffusion probabilistic model optimized for medical image-to-image generation^31^. Fast-DDPM reduces both training and inference time by aligning training and sampling steps and using fewer time steps.

Beyond GAN and diffusion-based models, several recent works explore alternative generative frameworks for medical image transformation. Chen et al.^32^ propose Diffusion Prior Synthesis and Optimization (DPSO), a source-free diffusion framework that leverages a probability flow ODE and target-domain priors to synthesize cross-modality MR images without access to source-domain data. UniSyn^33^ is a foundation-style generative model that synthesizes multiple imaging modalities conditioned on metadata, enabling flexible multi-modal image generation. Yazdani et al. propose a flow-matching–based generative framework for efficient and high-quality medical image synthesis^34^. The method learns continuous transport maps between noise and data distributions via flow matching, allowing faster one-step or few-step sampling. Applied to MRI synthesis tasks, the approach aims to achieve comparable or superior image quality and structural consistency relative to diffusion-based models while reducing inference time.

While the majority of the studies described above utilize conditional generative techniques, they often stop short of taking full advantage of the generative process. In most cases, for a given input image, they generate a single output image and treat this as the prediction. For example, the residual vision transformer (ResViT) model presented in^35^ is built based on the probabilistic formulation (GAN-based framework); however, in practice, it is used deterministically. We include this model in our comparisons as a benchmark due to the high performance of vision transformers reported in medical imaging tasks^36^. See Section ResViT for a brief explanation of this model. In contrast to this, in this work, we treat the generative algorithms as tools that allow us to sample from a conditional distribution and for a single input image generate an ensemble of output images. Thereafter, we use these images to generate our best guess for the output image and also quantify the pixel-wise uncertainty in this guess. We also demonstrate that a measure of this uncertainty can be used to determine whether the input image is tainted with artifacts or contains brain pathologies and is, therefore, out of distribution (OOD). We believe that the ability to detect anomalous input images without any supervised training will be an important component of a quality control process for image transformation. We also note that while a few prior works acknowledge the ability of generative models to produce multiple plausible outputs^37,38^, they typically do not analyze the resulting sample distributions or apply them to downstream tasks such as uncertainty quantification. Meanwhile, most existing approaches to uncertainty quantification in medical imaging rely on Bayesian inference or model ensembles^39–41^ and only a few rare studies, such as^42^, address uncertainty in the specific context of MR image transformation. In contrast, our work directly leverages the generative sample distribution to compute voxel-wise uncertainty and demonstrates its practical utility for detecting out-of-distribution inputs in brain MR images.

In the current study, we also address a critical gap in the current understanding of generative models employed for transforming brain MR images. We implement and test three promising models, namely the conditional GAN (cGAN)^43–46^, a diffusion model based on the conditional noise-conditional score network (NCSN)^20–22^, and the conditional denoising diffusion probabilistic model (DDPM)^47,48^, and systematically compare their performance on two benchmark datasets. Through this comparison, we note that the diffusion models yield the most accurate results, followed by a deterministic Direct model based on the U-Net architecture, cGAN model, and finally the ResViT model. We believe that these results will help guide future practitioners in selecting appropriate models for their tasks.

The remainder of the manuscript is organized as follows. In the following section, we present results for the three generative models and two benchmark models applied to brain MR image transformation tasks. Thereafter, we discuss the performance of these algorithms and explicitly demonstrate the benefits of treating the image transformation problem as a problem of conditional image generation. The benefits include more accurate results and the ability to detect OOD input images. Thereafter, we describe in detail the models implemented and tested in this study, the datasets used for training and testing the models, and the metrics used for evaluating their performance. We end the manuscript with conclusions and remarks for future work.

## Results

**Fig. 1:**
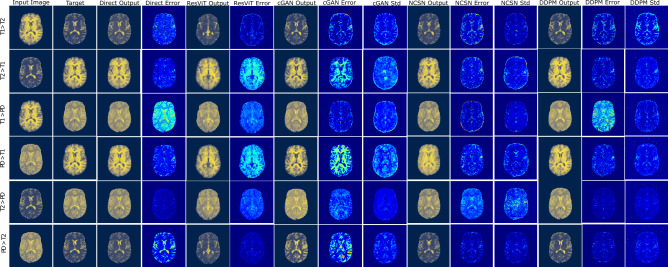
MR image transformation for an axial slice image from the IXI dataset. Column 1: input image; column 2: target image; columns 3-4: predicted and error images for the direct model; columns 5-6: predicted and error images for the ResViT model; columns 7-9: predicted, error, and standard deviation images for cGAN; columns 10-12: predicted, error and standard deviation images for NCSN; columns: 13-15: predicted, error and standard deviation images for DDPM.

We implement a direct inference model based on a U-Net architecture (called the Direct model in this work) that solves the deterministic image transformation problem and is trained using a mean squared error loss. We also utilize a residual vision transformer (ResViT) model as a benchmark. Moreover, we implement a conditional Wasserstein GAN (cGAN), which comprises a generator and critic that are trained together using a Wasserstein loss. In addition, we implement two score-based diffusion models. These are the conditional versions of the noise-conditional score network (NCSN) model and the denoising diffusion probabilistic (DDPM) model. Both these models learn to transform samples generated from a zero-mean uncorrelated high-dimensional Gaussian distribution to samples from the desired conditional distribution of images by learning and using the score function for this transformation. They differ from each other in the variance of the Gaussian distribution. The NCSN model uses a distribution with a large variance, whereas the DDPM model uses a distribution with unit variance. All the models are trained and tested using the IXI^49^ (Information eXtraction from Images project) and OASIS^50^ (Open Access Series of Imaging Studies) datasets that include T1, T2, proton density (PD), and fluid-attenuated inversion recovery (FLAIR) images for healthy subjects and patients with Alzheimer’s disease. The probabilistic models generate multiple images for a single input image. For these models, the pixel-wise mean image obtained from these samples is used as the best guess, and the pixel-wise standard deviation image is used as a measure of uncertainty in the transformation. The performance of all models is evaluated against the ground truth by computing metrics like the structural similarity index (SSIM), peak signal-to-noise ratio (PSNR), learned perceptual image patch similarity (LPIPS)^51^, and boundary SSIM (BSSIM) between the ground truth and transformed images. Furthermore, the slice consistency SSIM (SCSSIM) metric is used to assess the consistency of the models in generating adjacent slices. These aspects of the work are described in detail in the Methods section.

**Fig. 2:**
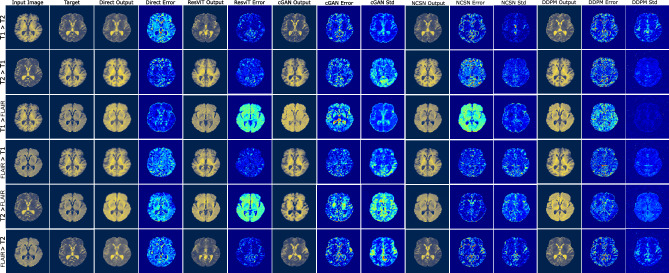
MR image transformation for an axial slice image from the OASIS dataset. Column 1: input image; column 2: target image; columns 3-4: predicted and error images for the direct model; columns 5-6: predicted and error images for the ResViT model; columns 7-9: predicted, error, and standard deviation images for cGAN; columns 10-12: predicted, error and standard deviation images for NCSN; columns: 13-15: predicted, error and standard deviation images for DDPM.

In Figs. 1 and 2, we present typical results for all possible image transformation types within the IXI and OASIS datasets, respectively. Each dataset contains three different image types, and we transform images from one type to the other two. Consequently, in each figure, we present results that are organized in six rows based on the type of transformation. From left to right, in the first and second columns, we present the input and ground truth (labeled as target) images, respectively. In the remaining columns, we present model outputs. The third and fourth columns contain the predicted image and the error for the Direct model. Here, the error is defined as the absolute value of the pixel-wise difference between the target and the prediction. The fifth and sixth columns belong to the prediction and error of the ResViT model. The seventh to ninth columns contain the prediction, error, and pixel-wise standard deviation images for the cGAN model. Finally, columns 10-12 and 13-15 contain the corresponding results for the NCSN and DDPM models, respectively.

**Fig. 3:**
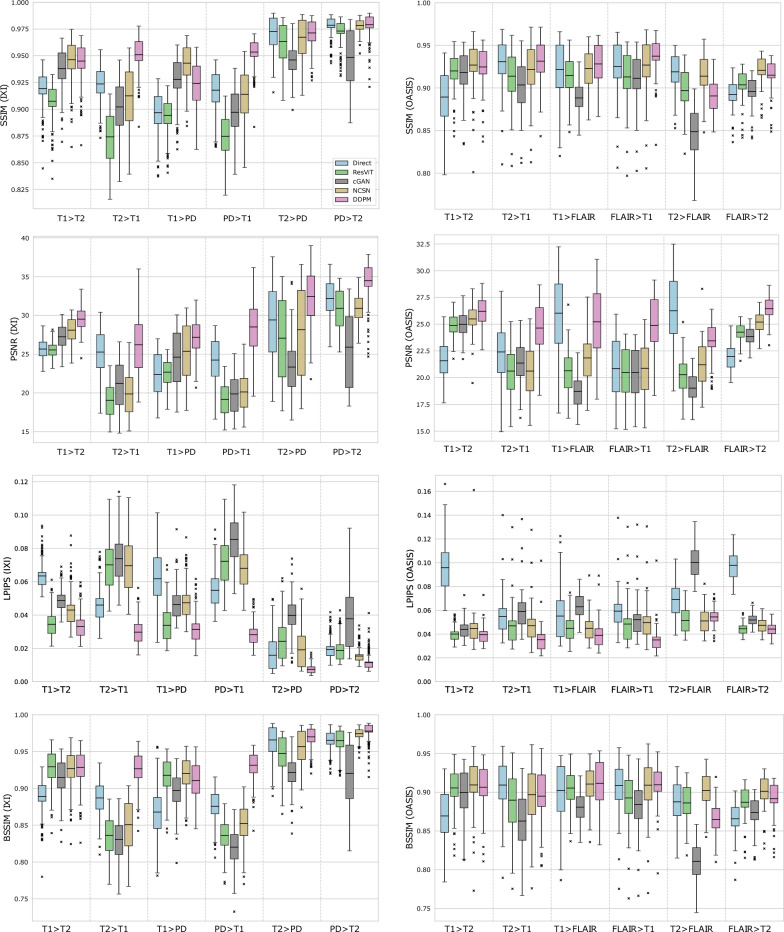
Boxplots of SSIM, PSNR, LPIPS, and BSSIM metrics for the IXI (left) and OASIS (right) datasets for the Direct (blue), ResViT (green), cGAN (gray), NCSN (brown), and DDPM (pink) models. Each plot contains results for the six distinct image transformation types.

For each image in the test set, we evaluate the SSIM, PSNR, LPIPS, and BSSIM metrics between the predicted and target image and present the statistics in Fig. 3. For SSIM, PSNR, and BSSIM metrics, higher values indicate a closer match between the target and predicted images, while for LPIPS, lower values indicate better similarity. For SSIM and BSSIM, a value of 1, and for LPIPS, a value of 0, indicates a perfect match. Results for the IXI dataset are presented in the four left plots, while those for the OASIS dataset are presented in the right plots. Within each plot, we present results for all five models applied to six different transformation types in the form of a box plot. The rectangular extent of each box displays the interquartile range (IQR) where $50\%$ of the values reside, and the whiskers depict the range of the values excluding the outliers. The outliers are displayed as markers in the plots and are defined as values that are smaller than 1.5 IQR below the $25^{th}$ percentile and greater than 1.5 IQR above the $75^{th}$ percentile.

In Table 1, we present the numerical values of the SSIM, PSNR, LPIPS, and BSSIM for test subjects from the IXI and OASIS datasets. Each row contains numerical values (the average value and the standard deviation in parentheses) of the metrics. We used pairwise one-sided Wilcoxon signed-rank tests ($p < 0.05$) to assess statistical significance in the reported metrics. A model is considered the best if it performs significantly better than all other models, allowing multiple models to be considered best when differences between them are not significant. The best-performing models are highlighted in bold in Table  1 (also in Tables  2,  4, and  5). For each row, we highlight the model or models with the highest value of each similarity metric. For SSIM, we observe that out of the twelve transformations, the DDPM model is the most accurate in six cases, the NCSN is the most accurate in four cases, and the Direct model is the most accurate in two cases. For the PSNR metric, the DDPM and Direct models outperform other models in ten and two cases, respectively. When focusing on the LPIPS metric, the DDPM method yields the highest values in eleven out of twelve cases, and NCSN is the best in one case. For the BSSIM metric, DDPM performs best in six cases, followed by NCSN in four, and Direct and ResViT models each leading in one case. The cGAN model never achieves the highest value for BSSIM in any transformation.

**Table 1:** Statistics (mean and standard deviation in parentheses) of SSIM, PSNR, LPIPS, and BSSIM metrics for test subjects from IXI and OASIS datasets for Direct, ResViT, cGAN, NCSN, and DDPM models. Each row lists the metrics for the indicated transformation, with the best results (the highest for SSIM, PSNR, and BSSIM and the lowest for LPIPS) highlighted in bold font.

	Direct	ResViT	cGAN	NCSN	DDPM
SSIM: IXI					
$T1 \,\, T2$	91.93 (1.67)	90.75 (1.76)	93.80 (1.91)	**94.62 (1.95)**	**94.50 (1.83)**
$T2 \,\, T1$	92.36 (1.72)	87.42 (2.42)	90.22 (2.37)	91.26 (2.90)	**95.10 (1.61)**
$T1 \,\, PD$	89.68 (1.93)	89.42 (1.66)	92.79 (2.17)	**94.31 (1.80)**	92.42 (2.06)
$PD \,\, T1$	91.79 (1.91)	87.47 (2.05)	89.72 (2.13)	91.41 (2.41)	**95.35 (1.28)**
$T2 \,\, PD$	**97.26 (1.56)**	96.32 (1.78)	94.61 (1.51)	96.73 (1.87)	**97.13 (1.35)**
$PD \,\, T2$	97.85 (0.93)	97.31 (1.16)	94.84 (2.70)	97.83 (0.62)	**97.91 (1.17)**
SSIM: OASIS					
$T1 \,\, T2$	88.94 (3.27)	92.00 (2.55)	91.82 (2.80)	**92.69 (3.03)**	92.47 (2.71)
$T2 \,\, T1$	**93.08 (3.06)**	91.39 (3.23)	90.35 (3.02)	92.15 (3.22)	**93.15 (2.63)**
$T1 \,\, FLAIR$	**92.15 (3.71)**	91.45 (2.39)	88.83 (2.16)	92.29 (2.24)	**92.82 (2.59)**
$FLAIR \,\, T1$	92.52 (3.26)	91.29 (3.26)	91.14 (3.08)	92.69 (3.33)	**93.73 (2.33)**
$T2 \,\, FLAIR$	**91.89 (2.48)**	89.69 (2.57)	84.86 (3.06)	**91.38 (2.47)**	89.06 (2.13)
$FLAIR \,\, T2$	89.24 (1.83)	90.36 (1.93)	89.58 (1.77)	**92.08 (1.90)**	91.50 (2.02)
PSNR: IXI					
$T1 \,\, T2$	25.66 (1.18)	25.55 (0.96)	27.25 (1.54)	28.10 (1.67)	**29.52 (1.42)**
$T2 \,\, T1$	25.28 (2.88)	19.07 (2.14)	21.23 (3.02)	19.87 (2.79)	**26.22 (3.67)**
$T1 \,\, PD$	22.39 (2.63)	22.68 (1.72)	24.61 (3.31)	25.37 (3.48)	**27.18 (2.35)**
$PD \,\, T1$	24.25 (2.92)	19.16 (2.07)	19.87 (2.61)	20.13 (2.54)	**28.51 (3.47)**
$T2 \,\, PD$	29.42 (4.72)	27.07 (5.06)	23.34 (3.28)	28.15 (5.71)	**32.46 (3.33)**
$PD \,\, T2$	32.21 (2.39)	30.93 (2.53)	25.90 (4.71)	30.93 (1.79)	**34.48 (2.50)**
PSNR: OASIS					
$T1 \,\, T2$	21.58 (1.83)	24.87 (1.19)	24.99 (1.28)	25.49 (1.52)	**26.20 (1.41)**
$T2 \,\, T1$	22.40 (2.87)	20.61 (2.30)	21.36 (1.94)	20.62 (2.38)	**24.64 (2.38)**
$T1 \,\, FLAIR$	**26.03 (3.38)**	20.64 (2.17)	18.71 (1.66)	21.84 (2.15)	**25.22 (3.19)**
$FLAIR \,\, T1$	20.85 (2.64)	20.49 (2.33)	20.49 (2.28)	20.88 (2.56)	**24.87 (2.59)**
$T2 \,\, FLAIR$	**26.26 (3.26)**	20.28 (1.91)	19.01 (1.38)	21.21 (2.19)	23.44 (1.96)
$FLAIR \,\, T2$	22.00 (1.32)	24.25 (0.91)	23.84 (0.83)	25.20 (1.05)	**26.45 (1.12)**
LPIPS: IXI					
$T1 \,\, T2$	0.063 (0.008)	0.034 (0.008)	0.049 (0.006)	0.043 (0.010)	**0.033 (0.008)**
$T2 \,\, T1$	0.046 (0.011)	0.070 (0.016)	0.074 (0.014)	0.070 (0.018)	**0.030 (0.007)**
$T1 \,\, PD$	0.062 (0.016)	**0.034 (0.010)**	0.046 (0.010)	0.047 (0.011)	**0.031 (0.009)**
$PD \,\, T1$	0.055 (0.011)	0.072 (0.014)	0.085 (0.014)	0.068 (0.013)	**0.028 (0.007)**
$T2 \,\, PD$	0.016 (0.010)	0.024 (0.012)	0.040 (0.010)	0.019 (0.011)	**0.007 (0.003)**
$PD \,\, T2$	0.019 (0.006)	0.019 (0.007)	0.038 (0.019)	0.015 (0.004)	**0.011 (0.005)**
LPIPS: OASIS					
$T1 \,\, T2$	0.096 (0.022)	**0.040 (0.007)**	0.044 (0.009)	0.045 (0.017)	**0.040 (0.008)**
$T2 \,\, T1$	0.055 (0.017)	0.047 (0.018)	0.059 (0.017)	0.047 (0.017)	**0.035 (0.012)**
$T1 \,\, FLAIR$	0.055 (0.022)	0.045 (0.011)	0.063 (0.012)	0.045 (0.011)	**0.039 (0.013)**
$FLAIR \,\, T1$	0.059 (0.017)	0.048 (0.018)	0.052 (0.017)	0.050 (0.017)	**0.035 (0.012)**
$T2 \,\, FLAIR$	0.069 (0.016)	0.052 (0.011)	0.100 (0.013)	**0.051 (0.011)**	0.054 (0.008)
$FLAIR \,\, T2$	0.098 (0.013)	**0.045 (0.005)**	0.052 (0.005)	0.047 (0.006)	**0.044 (0.005)**
BSSIM: IXI					
$T1 \,\, T2$	88.91 (2.30)	**92.94 (2.58)**	91.45 (2.53)	92.69 (2.58)	**92.84 (2.57)**
$T2 \,\, T1$	88.70 (2.32)	83.60 (2.80)	83.06 (2.80)	85.07 (3.54)	**92.66 (2.30)**
$T1 \,\, PD$	86.79 (3.43)	91.77 (2.44)	89.72 (2.54)	**92.01 (2.32)**	91.06 (2.61)
$PD \,\, T1$	87.58 (2.21)	83.58 (2.29)	82.02 (2.52)	85.21 (2.75)	**93.14 (1.95)**
$T2 \,\, PD$	**96.57 (2.09)**	94.72 (2.46)	92.17 (2.16)	95.68 (2.43)	**96.99 (1.41)**
$PD \,\, T2$	96.53 (1.33)	96.50 (1.65)	92.02 (4.42)	97.41 (0.77)	**97.78 (1.25)**
BSSIM: OASIS					
$T1 \,\, T2$	86.93 (3.47)	90.56 (3.01)	89.97 (3.31)	**90.94 (3.56)**	90.64 (3.17)
$T2 \,\, T1$	**90.92 (3.72)**	88.98 (3.85)	86.28 (3.75)	89.68 (3.82)	89.50 (3.61)
$T1 \,\, FLAIR$	**90.23 (4.04)**	90.54 (2.49)	88.07 (2.22)	**91.05 (2.49)**	**91.14 (3.22)**
$FLAIR \,\, T1$	**90.85 (3.44)**	89.26 (3.60)	88.43 (3.36)	**90.91 (3.63)**	**90.96 (2.59)**
$T2 \,\, FLAIR$	88.76 (2.95)	88.61 (2.53)	81.07 (2.53)	**90.23 (2.34)**	86.47 (2.38)
$FLAIR \,\, T2$	86.58 (2.19)	88.71 (2.27)	87.38 (2.06)	**90.11 (2.32)**	89.17 (2.36)

In addition to exploring the performance of MR image transformation models using inputs from intra-dataset test subjects, we discuss how different models respond to input images outside of the distribution for which they were trained. We refer to these input images as out-of-distribution (OOD) images and aim to explore how the models respond to these inputs and the possibility of detecting these inputs using the generative property of the probabilistic models. For this purpose, we utilize several datasets with OOD inputs. In the first experiment, we pass T1 images from the OASIS test subjects to the T1 to T2 transformation models trained on the IXI dataset. We compare the T2 output images against the available targets using SSIM, PSNR, LPIPS, and BSSIM metrics. We show the average results in Table 2, where each row belongs to a metric. The numbers in the parentheses indicate the changes compared to the results derived from a model trained and evaluated on the OASIS dataset. As expected, we observe that the majority of models demonstrate a decrease (indicated by a negative sign for SSIM, PSNR, and BSSIM and a positive sign for LPIPS) in their performance when transforming MR images using OOD inputs. We also observe that the highest decrease in the SSIM metric occurs in the NCSN, suggesting the possibility of a lower generalizability in these models. On the other hand, the ResViT model demonstrates the lowest changes in the SSIM and BSSIM metrics. This can stem from the fact that the ResViT model benefits from a sub-model that is pre-trained using a gigantic medical imaging dataset, facilitating higher generalizability across inputs. We further notice that LPIPS shows minimal changes compared to other metrics for most models.

**Table 2:** Average calculated similarity metrics for T1 to T2 transformations using the model trained on the IXI dataset but evaluated on test subjects from the OASIS dataset (OOD inputs). The values in the parentheses show the changes in the performance compared to the models that are also trained on the OASIS dataset. A negative value for SSIM, PSNR, and BSSIM and a positive value for LPIPS indicate that the performance is lower when using OOD inputs and vice versa.

	Direct	ResViT	cGAN	NCSN	DDPM
SSIM	85.86 (-3.08)	**91.05** (-0.95)	89.02 (-2.80)	88.43 (-4.26)	**91.04** (-1.43)
PSNR	22.97 (+1.39)	24.12 (-0.75)	23.60 (-1.39)	24.38 (-1.11)	**25.24** (-0.96)
LPIPS	0.080 (-0.016)	**0.050** (+0.010)	0.071 (+0.027)	0.081 (+0.036)	0.054 (+0.014)
BSSIM	82.11 (-4.82)	** 89.26** (-1.30)	86.40 (-3.57)	86.18 (-4.76)	88.48 (-2.16)

**Fig. 4:**
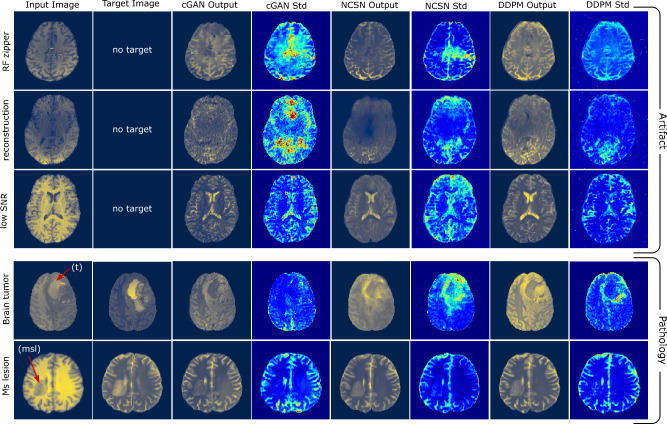
Response of models to out-of-distribution (OOD) input images. Column 1: input image; column 2: target image (when available); column 3-4: output and standard deviation images for the cGAN model; columns 5-6: transformed image and standard deviation for NCSN; columns 7-8: transformed image and standard deviation for DDPM. Rows 1, 2, and 3: images with RF zipper, reconstruction artifact, and low SNR; rows 4-5: images with brain tumor and white matter MS lesion pathologies.

In another experiment, we use sample input MR images with artifacts and brain pathologies (MS lesions and brain tumors) from three pubic datasets discussed in Datasets and Pre-processing. We show several examples of the outputs of the models using OOD inputs in Fig. 4. In this figure, from left to right, the first and second columns belong to the input and target images, where the target image is only available for images with pathology, as images with artifacts do not have T2 ground truth images. The third, fifth, and seventh columns represent the output images, and the fourth, sixth, and eighth columns show the standard deviation images, respectively, for the cGAN, NCSN, and DDPM models. Each row belongs to the input image with the indicated MR image anomaly. The first, second, and third rows correspond to images with artifacts: a strong RF zipper artifact (dashed-line shaped strong signals), an MR image reconstruction artifact, and a low SNR issue. We observe that all three models produce less reliable results when using images outside the distribution of the training dataset. We note that the Direct and ResViT models also yield inaccurate results, which are not shown here for brevity. We also observe that the probabilistic models exhibit responses with different characteristics to these inputs in their standard deviation images. We particularly see an increase in the standard deviation values, especially for the cGAN and NCSN models. While OOD inputs are more likely to result in inaccurate outputs, the information derived from the standard deviation images can be utilized to detect these potential inaccuracies, an option that is not available for deterministic models. The next row belongs to an image with a brain tumor marked as (t). As can be seen in the target column, the tumor area yields a hyperintense signal region in the target T2 image. However, the models reconstructed the tumor as a hypo-intense area in their outputs. This inaccuracy stems from the fact that the images with tumors are not included in the training data. In this example, the cGAN model does not demonstrate an increase in the standard deviation in the areas with the tumor. In contrast, the standard deviation is significantly higher in a relatively large area around the tumor for the NCSN model. The DDPM model shows an increase in the standard deviation for the areas close to the tumor. In the last row, the input image contains a white matter lesion marked as (msl). As can be seen, all three models have been relatively successful in reconstructing the lesion area and present slightly higher standard deviations almost globally.

The probabilistic models allow the evaluation of the pixel-wise standard deviation in the transformed images, which can be considered as a measure of uncertainty. It is worth considering whether this measure of uncertainty depends on whether the input image is OOD. From Fig. 4, we observe that this might be the case since the standard deviation in the transformed images for OOD input images is typically larger than the standard deviation for normal images.

**Fig. 5:**
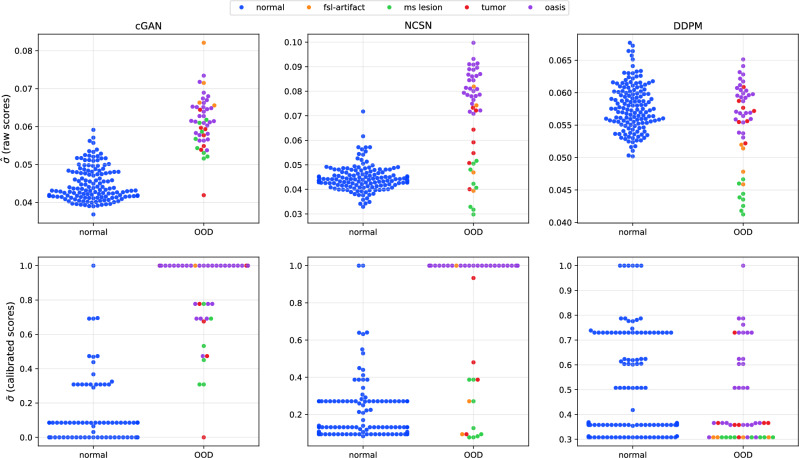
Distribution of $\hat{\sigma }$, a measure calculated from the standard deviation images, for cGAN, NCSN, and DDPM models. Distributions are divided into left: normal input images and right: out-of-distribution (OOD) or images with pathology or artifact, as indicated with different colors in the legend. The first and second rows show the score values before and after calibration using the isotonic regression method.

To explore the dependency of the standard deviation values on OOD inputs, we introduce a scalar score $\hat{\sigma }$ calculated as the value of pixel-wise standard deviation averaged over all voxels of the brain for a given 3D image volume. The values of raw scores $\hat{\sigma }$ are demonstrated as swarm plots in three sets shown in the first row of Fig. 5 for cGAN, NCSN, and DDPM models. In each plot, the left column (normal) contains results obtained by transforming normal images from 144 IXI test subjects and evaluating $\hat{\sigma }$ for each subject using the T1 to T2 transformation model trained on the IXI training dataset. The left columns (OOD) contain calculated $\hat{\sigma }$ for subjects with artifacts (shown in orange), those with MS lesions (in green), and those with brain tumors (in red), from which five examples are displayed in Fig. 4. This column also shows results for input images from the OASIS dataset, which are considered OOD, as the model is trained on the healthy subjects from the IXI dataset, but the OASIS dataset contains subjects with Alzheimer’s and those in the aging stages. From Fig. 5, we observe that a large value of $\hat{\sigma }$ is often an indicator of OOD input images for the cGAN and NCSN models. We also observe that this conclusion can not be generalized to $\hat{\sigma }$ values calculated for the DDPM model.

**Fig. 6:**
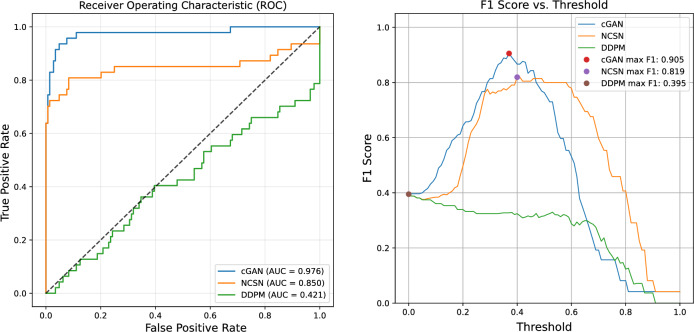
Left: ROC curves comparing the classification performance of cGAN, NCSN, and DDPM models based on the raw calculated average standard deviation $\hat{\sigma }$ for detecting normal from OOD images. The closer the curve is to the top-left corner, the better the model. Right: F1 score curve for different threshold values. A high F1 score indicates that the model has both high precision and high recall, meaning it makes few false positives and false negatives.

These are further examined by using $\hat{\sigma }$ as a score to classify the input images as either normal or OOD. We do this by varying the threshold for this classification to generate a receiver operating characteristic (ROC) curve and computing the area under this curve (AUC). This is shown in the left part of Fig. 6 for $\hat{\sigma }$ values. Recall that for a perfect classifier, AUC is equal to 1, whereas an AUC of 0.5 corresponds to performance no better than a random guess. As shown in the plot legends, the AUC for the cGAN, NCSN, and DDPM models are respectively 0.976, 0.850, and 0.421. These results indicate that the scores calculated from the outputs of the cGAN and NCSN models are effective in classifying input images as normal or OOD. The right plot in Fig. 6 presents the F1 scores computed to evaluate the classification performance based on the normalized uncertainty values $\hat{\sigma }$ for the cGAN, NCSN, and DDPM models, where the maximum achievable F1 scores, as indicated in the legend, are 0.905, 0.819, and 0.395. The F1 score serves as a balanced metric that combines both precision and recall (See Evaluation metrics and statistical measures for the definition).

Despite the strong discriminative performance of $\hat{\sigma }$ scores in separating normal and OOD inputs, further analyses revealed that these values were not well-calibrated for the binary classification task. This was reflected in higher error values for the expected calibration error (ECE), Brier score, and negative log-likelihood (NLL) shown in the first three columns of Table 3 (see Evaluation metrics and statistical measures for definitions). We applied isotonic regression calibration to the scalar OOD scores $\hat{\sigma }$ produced by the cGAN, NCSN, and DDPM models. Calibration was performed on the validation set, using five-fold cross-validation on the in-distribution (ID) data combined with a randomly selected subset of OOD samples to ensure balanced coverage of both classes. This procedure learns a monotonic mapping that adjusts raw uncertainty estimates ($\hat{\sigma }$) so that their magnitudes better reflect empirical probabilities of belonging to the OOD class. The resulting calibration models were subsequently applied to rescale the OOD scores of the unseen test data, ensuring that all reported reliability metrics correspond to held-out samples. As shown in the second part of Table 3, the calibrated scores, denoted as ($\bar{\sigma }$), yielded substantially lower ECE, NLL, and Brier score values, with only a slight reduction in AUC. The calibrated $\bar{\sigma }$ distributions, shown in the second row of Fig. 5, demonstrate probabilistically meaningful separations between in-distribution and OOD inputs, making calibrated scores more generalizable and interpretable.

**Table 3:** Performance metrics for binary classification of normal versus out-of-distribution OOD input images based on the normalized calculated mean of standard deviation $\hat{\sigma }$ (left) and the score values after calibration using the isotonic regression method (right). The best value in each row (the highest for AUC and the lowest for the other metrics) is highlighted using bold font.

	normalized scores ($\hat{\sigma }$)			calibrated scores		
	cGAN	NCSN	DDPM	cGAN	NCSN	DDPM
AUC	**0.976**	0.850	0.421	0.973	0.843	0.438
ECE	0.182	0.169	0.449	**0.043**	0.088	0.249
Brier score	0.218	0.214	0.222	**0.044**	0.092	0.283
NLL	0.723	0.698	0.760	**0.256**	0.437	1.129

## Discussion

In the first part of this section, we investigate the discussed models in terms of their quantitative accuracy and the qualitative characteristics of their outputs. In the second part, we briefly provide examples of the neuroimaging implications and considerations that need to be taken into account when utilizing these models.

Images from the IXI dataset are of better quality and display less heterogeneity when compared with images from the OASIS dataset. Some images in the OASIS dataset suffer from low contrast and poor image quality. Consequently, on average, all image transformation methods perform better on the IXI dataset. This is seen clearly by computing the average SSIM, PSNR, LPIPS, and BSSIM values for each method over all six transformation types for the two datasets. These values are reported in Table 4, where we observe that for every method, all metrics are higher for the IXI dataset.

**Table 4:** Average similarity metrics (SSIM, PSNR, LPIPS, and BSSIM) for the Direct, ResViT, cGAN, NCSN, and DDPM methods. Averages are calculated over all six transformation types for all test subjects in the IXI and OASIS datasets. The last three rows, labeled “COMBINED”, represent the average metrics for each method across both datasets and all transformations.

		Direct	ResViT	cGAN	NCSN	DDPM
IXI	SSIM	93.48	91.45	92.66	94.36	**95.40**
	PSNR	26.54	24.08	23.70	25.43	**29.73**
	LPIPS	0.044	0.042	0.055	0.044	**0.024**
	BSSIM	90.85	90.52	88.41	91.35	**94.08**
OASIS	SSIM	91.30	91.03	89.43	**92.21**	**92.12**
	PSNR	23.19	21.86	21.40	22.54	**25.14**
	LPIPS	0.072	0.046	0.062	0.047	**0.041**
	BSSIM	89.05	89.44	86.87	**90.49**	89.65
COMBINED	SSIM	92.87	91.33	91.76	93.76	**94.48**
	PSNR	25.60	23.46	23.06	24.62	**28.44**
	LPIPS	0.052	0.043	0.057	0.045	**0.029**
	BSSIM	90.34	90.22	87.98	91.11	**92.84**

We note that while the outputs of the models are generated as batches of 2D slices, they are then stacked to reconstruct 3D volumes. All evaluation metrics (SSIM, PSNR, LPIPS, and BSSIM) in this work are computed based on normalized 3D volumes rather than slice-wise averages to ensure that the evaluation metrics reflect any potential inconsistencies across slices. To further assess volumetric coherence, we introduced a slice-consistency metric, SCSSIM, that measures the stability of inter-slice similarity across the reconstructed volume (see Evaluation metrics and statistical measures section for definition). The computed values are presented in Table 5. As shown, all methods achieved high slice-consistency scores (ranging from 97.20 to 99.25), indicating that the models preserve spatial continuity across slices.

**Fig. 7:**
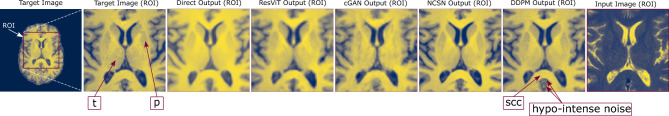
T2 to T1 transformation from the IXI test dataset. From left to right: target T1 image with a square-shaped region of interest (ROI), followed by images of the ROI for the target image, transformed images generated by the Direct, ResViT, cGAN, DDPM, and NCSN models, and the input image. In the ROI target image, the thalamus (t) and putamen (p), and in the DDPM image, the splenium of the corpus callosum (scc) and two hypo-intense pixels are marked by arrows.

From Table 4, we conclude that the cGAN model yields the lowest value for the PSNR, LPIPS, and BSSIM, and the second lowest for the SSIM metric. We speculate that the cause is the distortion observed at the boundaries of different types of brain tissues, as also reflected in low BSSIM values. These distortions are most clearly seen in the transformed T1 images. We show an example in Fig. 7, where for a test sample slice from the IXI dataset, we have plotted the target T1 image, a zoomed-in region of interest (ROI) in this image that encompasses the ventricles and basal ganglia, the corresponding transformed ROIs generated by the Direct, ResViT, cGAN, NCSN, and DDPM methods, and the ROI in the input T2 image. We will return to Fig. 7 in this section in order to illustrate qualitative trends observed in our study. As mentioned, in the cGAN output ROI of Fig. 7, we observe that the boundaries between subcortical structures like the thalamus and putamen (marked as (t) and (p) in the target image) and the white matter appear to be distorted in the transformed cGAN image. We note that these distortions are also observed in other images generated by the cGAN method. Our experience is consistent with other reports^17^ that claim that GAN-based models struggle to generate fine features and edges in transformed images. This is a significant drawback since it can lead to erroneous volumetric measurements in downstream tasks in MR imaging.

**Table 5:** Average slice consistency similarity metrics (SCSSIM) for the Direct, ResViT, cGAN, NCSN, and DDPM methods. Averages are calculated over all six transformation types for all test subjects in the IXI and OASIS datasets. The last row, labeled “COMBINED”, represents the average metrics for each method across both datasets and all transformations.

	Direct	ResViT	cGAN	NCSN	DDPM
IXI	99.09	98.84	98.77	99.10	**99.25**
OASIS	97.20	**98.12**	97.28	98.04	97.66
COMBINED	98.56	98.64	98.36	98.81	**98.80**

**Fig. 8:**
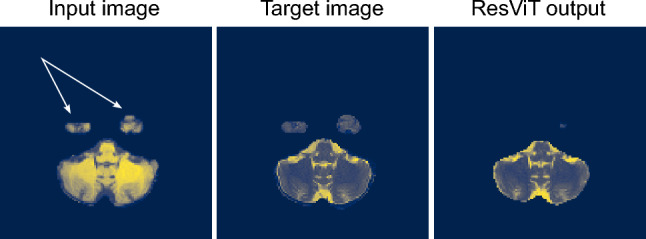
An example of false negative areas (shown using the white arrows in the input image), where parts of the brain are missing in the output of the ResViT model.

The model with the lowest SSIM and slightly higher PSNR and BSSIM is ResViT. We observe from Fig. 7 that, unlike cGAN, this model is successful in generating fine features and tissue boundaries. This can also be observed as a higher value of BSSIM for this model compared to the cGAN model. However, a closer look at atypical slices reveals that the ResViT model occasionally produces areas of false positives (cloud shapes outside the brain) and false negatives (missing part of the brain in the output). We note that the former is resolvable using appropriate post-processing. However, the latter is unrecoverable as part of the brain will be excluded from the output. We speculate that in patch-based hybrid models such as ResViT, small tissue islands can fall across patch boundaries, fragmenting their representation and making them prone to suppression during reconstruction. Improving spatial resolution, using overlapping or hierarchical patch embeddings^52,53^, and proper preprocessing, such as applying skull stripping after transformation, can mitigate this effect. We provide an example for this issue in Fig. 8, where the lower parts of the temporal lobes, where the white arrows are pointed in the input image, are missing in the output of the ResViT model.

The calculated metrics for the Direct model are relatively acceptable. However, visual inspection of the outputs suggests that this method generally produces transformed images that are blurry. From Fig. 7, we can clearly see that the ROI generated by the Direct method is blurry when compared with the target ROI and the ROIs generated by most other methods. We observe this trend for the Direct method for most transformation types and test samples. One possible explanation for this trend could be the use of the mean-squared error as a loss function. However, we found that this trend persisted when we changed the loss function to the mean absolute error function (results not shown here).

The DDPM model has the highest overall SSIM, PSNR, LPIPS, and BSSIM values, whereas the NCSN model yields the second-highest SSIM and BSSIM values. A visual inspection of the transformed images produced by these models in Fig. 7 reveals that both generate qualitatively accurate images. However, a close look at the image generated by the DDPM method indicates the presence of two hypo-intense pixels posterior to the splenium of the corpus callosum (marked as “scc” in the figure). We find these artifacts to be an occasional occurrence in the images generated by the DDPM method. As discussed in Supplementary Notes online, we speculate that this model exhibits a numerical instability for the MR image transformation problem that results in producing outlier pixels with extremely large or small (negative) values. The outlier values can be corrected by employing a denoising step described in the Methods section. However, extra care is needed when working with this model. We note that the similarity metrics for the DDPM method reported in Tables 1 and 4 are obtained after applying this denoising step.

In summary, considering SSIM as the primary quantitative metric, the DDPM model shows higher accuracy compared to NCSN (by $1.73\%$) as the second-best model. However, both models generate transformed images with comparable quality. Additionally, the former exhibits numerical instability, whereas the latter does not.

As we mentioned, the probabilistic formulation in the generative models allows a series of additional analyses that are not possible when working with deterministic models. Specifically, for any given pixel in the input images, we have a distribution of output values in the generated samples and a single ground truth value. Performing various analyses on univariate pixel-wise data can provide insights into the characteristics of these models and the distribution of their outputs. One of these aspects is whether the distribution of the outputs aligns with the observed target data. One way to assess this is through regression calibration plots^54^, which visualize the relationship between predicted confidence levels and the empirical coverage, i.e., the actual fraction of targets whose true values fall within the corresponding predicted confidence intervals. For example, whether 10% of the target values lie within the 10% confidence intervals calculated based on the samples generated by the model, and so on.

**Fig. 9:**
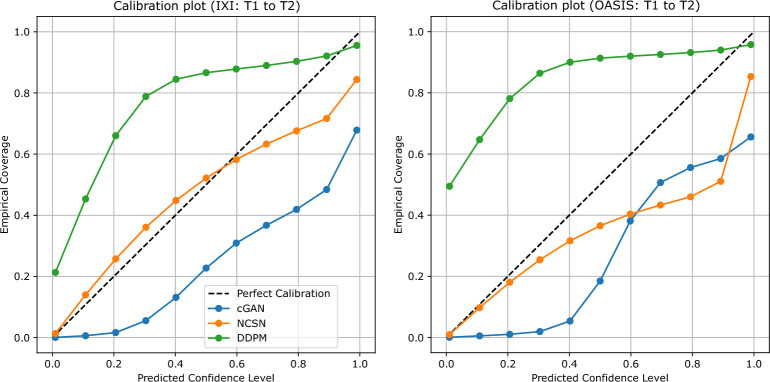
Regression calibration plot for T1 to T2 transformations using cGAN, NCSN, and DDPM models trained and evaluated on IXI (left) and OASIS (right) datasets.

We illustrate these regression calibration plots in Fig. 9 for the T1-to-T2 transformation of generative models on the IXI (left) and OASIS (right) datasets. A well-calibrated model produces a curve that closely follows the diagonal. Deviations below the diagonal indicate overconfident predictions (distributions of samples are too narrow), while curves above suggest underconfident predictions (distributions too wide). We note that the term “calibration” was also used in the context of binary classification of normal and OOD inputs from the standard deviation values. In the context of binary classification, the targets are labels with values of zero or one, and the scores form a distribution of values for the probability of belonging to the normal or OOD class. Here, in the context of regression calibration, the targets and outputs have continuous values. As shown, the orange curve, especially for the IXI dataset, is closest to the diagonal, indicating that the NCSN model is better calibrated compared to others. In contrast, the DDPM model tends to be underconfident, with true pixel values too often falling within the predicted intervals of samples. This suggests that the distributions of DDPM outputs are overly broad, reflecting standard deviations that are larger than what they should be. On the other hand, the cGAN model exhibits calibration curves that often lie below the diagonal, indicating overconfidence. This may arise from limited diversity in the generated outputs or from systematic bias in the predictions. We observed similar trends in transformations other than T1 to T2, which are not reported here for brevity.

The effectiveness of OOD detection based on the scores calculated from the standard deviation images is linked to model calibration. While NCSN uncertainty is well-calibrated and reliably captures ID variability, cGAN exhibits overconfident predictions for ID inputs but displays larger standard deviation image scores for OOD inputs. This behavior may arise from the adversarial training dynamics in cGAN, which amplify differences when encountering unfamiliar inputs. In contrast, DDPM produces broadly high uncertainty for all inputs, reducing the discriminative power of the scores for OOD detection. These observations highlight that both calibration quality and sensitivity to deviations determine how ensemble-derived uncertainties can be used to detect out-of-distribution images.

We provide a number of additional analyses based on the generative property of the cGAN, NCSN, and DDPM models in Supplementary Notes online. Specifically, we utilize the Shapiro-Wilk test to assess the normality of the distribution of the outputs. The results suggest that the outputs of the NCSN model demonstrate significantly higher normality compared to the other two models. We further investigate the impact of the number of samples on the accuracy of the outputs and the statistical measures, such as cumulants, in Supplementary Notes online.

**Fig. 10:**
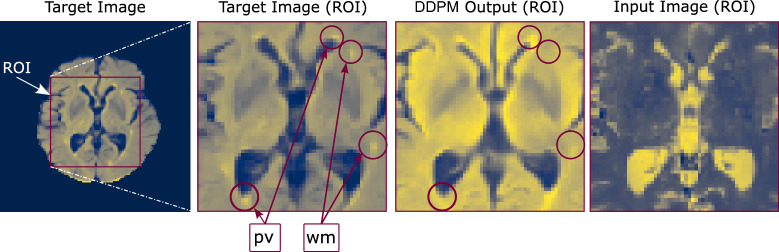
T2 to FLAIR transformation from the OASIS test dataset using the DDPM model. From left to right: the target FLAIR image, the ROI in the target image with white matter lesions (wm) and periventricular hyper-intense regions (pv), the ROI in the transformed image, and the ROI in the input T2 image.

In addition to quantitative evaluation and exploring the differences in the characteristics of the discussed models, recognizing the extent of their reliability is critical, especially in clinical contexts. For example, are models equally successful in all combinations of input and output contrasts, or are pathological features such as lesions preserved in the output images? To answer the first question, we note the differences when analyzing the metrics across all transformations in Table 1. For example, the DDPM model attains the two highest SSIM metrics when converting PD to T2 (97.91) and T2 to PD (97.13) images from the IXI dataset. This can be attributed to the inherent similarity in tissue contrast between these two MR image types, particularly in water-rich tissues. On the other hand, we observe the lowest SSIM when converting T2 to FLAIR (89.06) and FLAIR to T2 (91.50) images. One of the reasons for this drop in performance is the absence of certain features in the input image that are clearly observed in the target image. For example, when compared with the T2 images, FLAIR images are designed to demonstrate high sensitivity in highlighting white matter lesions and some other normal or pathological regions that appear as hyper-intense periventricular voxels. This is seen in Fig. 10, where in the target FLAIR image, we have marked several hyper-intense periventricular regions (pv) and white matter lesions (wm) that are very difficult to locate in the input T2 image. The transformed FLAIR image generated by the DDPM model is relatively successful in reconstructing periventricular hyper-intense regions, but fails to generate white matter lesions, as they are absent in the input image. We observe similar patterns in the output of other models.

**Fig. 11:**
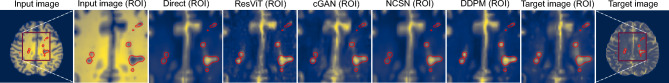
The results of T1 to T2 transformation of the discussed models for an input image with MS lesions. From left to right, the input image and the area within the highlighted ROI in the input image, the outputs of the indicated models, the target image within the ROI, and the target image are shown. The red curves indicate the manually segmented MS lesion areas.

We further conduct an experiment to investigate the ability of the models in preserving pathological features. We perform T1 to T2 image transformation using inputs that contain MS lesions from the MSSEG dataset. We illustrate an example of the outputs in Fig. 11. From left to right, the first and second images show the input image and the area within the highlighted ROI in the input image. The last and second-to-last images are the target images and the target image within the ROI. The remaining images (third to eighth) are the outputs of the models. The red curves indicate the MS lesion areas provided through manual segmentation by experts. As seen, most models have been relatively successful in reconstructing the lesions in their output. We quantify the ability of the models to preserve the lesions by calculating the Pearson correlation coefficient between the intensity of pixels within the lesion areas of the outputs and the target images. The results for the Direct, ResViT, cGAN, NCSN, and DDPM are respectively 0.748, 0.696, 0.669, 0.705, and 0.711. These numbers indicate a relatively strong correlation between the values of the output and target images within the lesion areas. This experiment presents an example of the successful preservation of pathologies in the output.

Another example is the effectiveness of the models in performing several image transformations on the OASIS test subjects that exhibit a range of pathologies, such as brain atrophy and Alzheimer’s disease lesions. However, we note that this is not always the case. We provided an example in Fig. 4, where the models fail to regenerate the brain tumor in their output. Therefore, beyond the extensive evaluations provided here, the selection of training data, the expectations from the outputs, and the evaluation strategies should be tailored based on the subsequent tasks. These are widely accepted points for any deep learning-based model in order to provide reliable outputs. However, the generative models hold the significance of providing tools that can warn the end-user when the outputs are more likely to be unreliable.

## Methods

### Problem formulation

We assume that a dataset of *N* paired MR images of an input and an output type is available. This dataset is denoted by $S=\{( \boldsymbol{x}^{(i)}, \boldsymbol{y}^{(i)})\}_{i=1}^N$, where $\boldsymbol{x}^{(i)} \in \mathbb {R}^{N_X}$ are images of the input type, $\boldsymbol{y}^{(i)} \in \mathbb {R}^{N_Y}$ are images of the output type, and $N_X$ and $N_Y$ are the number of pixels in the output and input images, respectively. In this study, $N_X=N_Y$ and each image is a two-dimensional axial image of the brain. It is further assumed that each image pair corresponds to the same physical slice and belongs to the same subject.

### Deterministic formulation (direct model)

In the deterministic formulation of the problem, given an input image, the aim is to solve a nonlinear regression problem to estimate the pixel intensity of the output image. To solve this problem, we assume that there exists a function, $\boldsymbol{f}:\mathbb {R}^{N_X} \longmapsto \mathbb {R}^{N_Y}$, that directly transforms the pixel intensity of the input image to the output image through a nonlinear transformation. We model $\boldsymbol{f}$ using a neural network and denote it by $\tilde{\boldsymbol{f}}$. This model is trained by minimizing a loss function that measures the difference between the output of the model and the target or ground truth for all images in the training dataset. This loss function is given by

1 $$\begin{aligned} \mathscr {L}(\tilde{\boldsymbol{f}})=\frac{1}{2 N}\sum _{i=1}^{N}|{\boldsymbol{y}}^{(i)} - \tilde{\boldsymbol{f}}( \boldsymbol{x}^{(i)})|^2. \end{aligned}$$

The trained model is $\tilde{\boldsymbol{f}}^*=\mathop {\mathrm {arg\,min}}\limits _{\tilde{\boldsymbol{f}}}\, \mathscr {L}(\tilde{\boldsymbol{f}})$ and is referred as the direct model. We use this model as a simple baseline and compare the performance of the probabilistic models against it. In order to make a fair comparison, a similar U-Net neural network architecture is used for all models, i.e., the direct model, the generator in the cGAN, the score network in the NCSN, and the denoiser in the DDPM. (see Supplementary Notes online for details.)

### Probabilistic formulation

In the probabilistic framework, we treat the input and output image types as random variables $\boldsymbol{X}$ and $\boldsymbol{Y}$, respectively. The dataset *S* represents samples drawn from the joint density function $p_{\boldsymbol{XY}}( \boldsymbol{x}, \boldsymbol{y})$. Using *S*, we aim to train deep generative models that receive a new input image $\boldsymbol{X} = \hat{\boldsymbol{x}}$ and can generate an ensemble of likely transformed generated images sampled from the conditional distribution, $\boldsymbol{y}^g \sim p_{\boldsymbol{Y}|\boldsymbol{X}}(\boldsymbol{y}|\hat{\boldsymbol{x}})$. These generated samples can be used to compute any desired statistic, including the sample mean, which is treated as the best guess for the inferred transformed image, and the sample standard deviation image, which is interpreted as a measure of uncertainty in the transformed image. We solve the probabilistic problem using three distinct generative models: conditional Wasserstein generative adversarial networks (cGAN), noise conditional score networks (NCSN), and denoising diffusion probabilistic models (DDPM) methods. These are described next.

### Conditional Wasserstein generative adversarial network (cGAN)

**Fig. 12:**
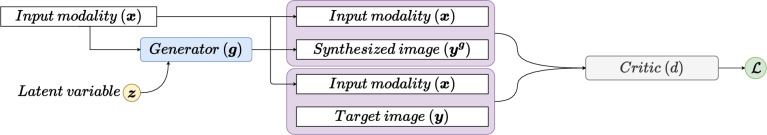
The conditional Wasserstein generative adversarial network (cGAN) architecture used in this work. The generator $\boldsymbol{g}$ receives the input image $\boldsymbol{x}$, and generates a synthesized image $\boldsymbol{y}^{\boldsymbol{g}}$ for any random latent vector $\boldsymbol{z}$. The critic *d* distinguishes between the generated synthesized image $\boldsymbol{y}^{\boldsymbol{g}}$ and target image $\boldsymbol{y}$ paired with $\boldsymbol{x}$. The training process involves updating $\boldsymbol{g}$ such that its outputs are indistinguishable from real images by *d* and updating *d* to become better at distinguishing the generated images from real ones.

As shown in Fig. 12, the cGAN architecture consists of two deep neural networks, a generator $\boldsymbol{g}$ and a critic or discriminator *d*. The generator, $\boldsymbol{g}:\mathbb {R}^{N_X} \times \mathbb {R}^{N_Z} \longmapsto \mathbb {R}^{N_Y}$, receives an input image $\boldsymbol{x}$ and an instance of the random latent vector $\boldsymbol{z} \in \mathbb {R}^{N_Z}$ and generates an output image $\boldsymbol{y}^g = \boldsymbol{g}( \boldsymbol{x}, \boldsymbol{z})$. The latent variable is sampled from a simple, known probability distribution, $p_{\boldsymbol{Z}}$. In this work, each component of the latent vector is independent and sampled from a standard normal distribution. By using a given input image $\boldsymbol{x}$ along with multiple instances of $\boldsymbol{z}$ drawn from $p_{\boldsymbol{Z}}$, the generator generates an ensemble of transformed images for a single input image. These images may be treated as samples drawn from a conditional distribution denoted by $\boldsymbol{y}^g \sim p^g_{\boldsymbol{Y}|\boldsymbol{X}}$.

The critic $d:\mathbb {R}^{N_X} \times \mathbb {R}^{N_Y} \longmapsto \mathbb {R}$ receives a pair of images and distinguishes between the pairs drawn from the true dataset, i.e., $( \boldsymbol{x}, \boldsymbol{y}) \sim p_{\boldsymbol{XY}}$, and pairs where the output image type is generated by the generator network, i.e., $( \boldsymbol{x}, \boldsymbol{y}^{\boldsymbol{g}})$, where $\boldsymbol{y}^{\boldsymbol{g}} \sim p_{\boldsymbol{Y}|\boldsymbol{X}}^{\boldsymbol{g}}$. This is accomplished by requiring the critic to yield larger values for images from the true dataset and smaller values for the images from the generated dataset. This leads to an adversarial loss function^44^ given by

2 $$\begin{aligned} \mathscr {L}({d},\boldsymbol{g})= \underset{\begin{array}{c} ( \boldsymbol{x}, \boldsymbol{y})\sim p_{\boldsymbol{XY}}\\ \boldsymbol{y}^{\boldsymbol{g}} \sim p^{\boldsymbol{g}}_{\boldsymbol{Y}|\boldsymbol{X}} \end{array}}{\mathbb {E}} [{d}( \boldsymbol{x}, \boldsymbol{y})-{d}( \boldsymbol{x}, \boldsymbol{y}^g )], \end{aligned}$$

and determining the optimal networks by solving a min-max optimization problem,

3 $$\begin{aligned} d^*(\boldsymbol{g})= & \mathop {\mathrm {arg\,max}}\limits _{ d}\, \left[ \mathscr {L}(d,\boldsymbol{g}) + \lambda \underset{\begin{array}{c} ( \boldsymbol{x}, \boldsymbol{y})\sim p_{\boldsymbol{XY}}\\ \boldsymbol{y}^{\boldsymbol{g}} \sim p^{\boldsymbol{g}}_{\boldsymbol{Y}|\boldsymbol{X}} \end{array}}{\mathbb {E}} [(\left\| \partial _{\mathfrak { \boldsymbol{y}}} {d}( \boldsymbol{x}, \hat{\mathfrak { \boldsymbol{y}}}) \right\| -1)^2]. \right] , \end{aligned}$$

4 $$\begin{aligned} \boldsymbol{g}^*= & \mathop {\mathrm {arg\,min}}\limits _{\boldsymbol{g}}\, \mathscr {L}(d^*(\boldsymbol{g}),\boldsymbol{g}). \end{aligned}$$

We note that the second term in Equation 3 is the gradient penalty term^45^, $\lambda$ is the penalty parameter, and $\hat{\mathfrak { \boldsymbol{y}}} =\epsilon \boldsymbol{y} +(1-\epsilon ) \boldsymbol{y}^{\boldsymbol{g}}$ where $\epsilon \sim \mathscr {U}(0,1)$. It can be shown that the optimal critic ($d^*$) measures the Wasserstein-1 distance between the true joint distribution and its generated counterpart^46^, and therefore the optimal generator ($\boldsymbol{g}^*$) aims to minimize this distance. This, in turn, implies that the generated conditional distribution $p_{\boldsymbol{Y}|\boldsymbol{X}}^g$ is close to the true conditional distribution $p_{\boldsymbol{Y}|\boldsymbol{X}}$.

We further note that the generator implemented in this work accepts $\boldsymbol{z}$ as input through a set of conditional instance normalization (CIN)^55^ blocks, as discussed in Supplementary Notes online. This is an example of the neural network considerations to incorporate stochasticity in generative models. In other words, a model can be constructed based on a probabilistic formulation while still lacking the appropriate design to generate multiple samples per input image. These models provide predictions in a deterministic fashion, a common practice in the literature, and therefore, lose any information derived from the distribution of generated images. See the ResViT model discussed next as an example.

### Residual vision transformation (ResViT)

The ResViT^35^ model also uses a generative adversarial network architecture, where the generator consists of a hybrid architecture of encoder, information bottleneck, and decoder. The encoder and decoder contain deep convolutional layers, and the information bottleneck consists of several stacked residual transformer (ART) blocks. Each ART block contains a transformer module, a CNN module, and external skip connections. The discriminator model contains several convolutional layers. When converting a single MR image type to another, the original implementation is designed to provide a single output (See Supplementary Notes online for details).

### Diffusion models

**Fig. 13:**
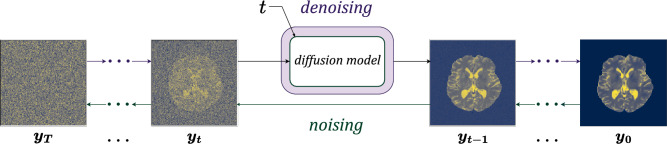
Forward noising and backward denoising processes in diffusion models. During training (from right to left) sample images go through several noising steps. This provides a series of images with different levels of noise. These images are used to train a model that receives a noisy image and time *t* and provides an output that is used to generate a less noisy image. After training, generating samples involves (from left to right) starting from a noise image and generating less noisy images, leading to the final generated image.

As illustrated in Fig. 13, the workflow of diffusion-based models (NCSN and DDPM) consists of a forward noising process (from right to left) and a backward denoising process (from left to right). In the unconditional version of a diffusion model, during the noising process, a sample is drawn from the true initial distribution $\boldsymbol{y}=\boldsymbol{y}_0 \sim p_0(\boldsymbol{y})$, i.e., an image from the training dataset, and an uncorrelated noise with variance $\beta _t=\sigma ^2_t,t = 1 \dots T$ is added to it. This process is repeated until the image looks like pure noise, i.e., $\boldsymbol{y}_T \sim \mathscr {N}\left( \boldsymbol{\mu }, \beta _T\mathbb {\boldsymbol{I}}\right)$, where $\mathbb {\boldsymbol{I}}\in \mathbb {R}^{N_Y}$ denotes the identity matrix. The data from the forward noising process is used to train a denoising neural network that receives a noisy image $\boldsymbol{y}_t \sim p_{t}(\boldsymbol{y})$ and generates an image $\boldsymbol{y}_{t-1} \sim p_{t-1}(\boldsymbol{y})$ with slightly less noise. This network is applied to an image drawn from the distribution of pure noise ${\boldsymbol{y}}_T \sim \mathscr {N}\left( \boldsymbol{\mu }, \beta _T \mathbb {\boldsymbol{I}} \right)$ in multiple denoising steps, $t = T \dots 1$, to generate a sample from $p_{0}(\boldsymbol{y})$. In the conditional version of this algorithm, the noising and denoising steps are conditioned on the input image $\boldsymbol{x}$.

### Noise conditional score network (NCSN)

The noising process in the NCSN method involves adding a noise to the initial image $\boldsymbol{y}_{0}$,

5 $$\begin{aligned} \boldsymbol{y}_{t} = \boldsymbol{y}_{0} + \sqrt{\beta _t} \boldsymbol{\epsilon }, \; t = 1 \dots T, \end{aligned}$$

where $\boldsymbol{\epsilon } \sim \mathscr {N}(0, \mathbb {\boldsymbol{I}}) \in \mathbb {R}^{N_Y}$, and the variances $\beta _t$ are hyper-parameters that are selected according to a schedule^21^. The training process in the NCSN learns a neural network model $\tilde{\boldsymbol{s}}(\boldsymbol{y},\boldsymbol{x},t): \mathbb {R}^{N_Y} \times \mathbb {R}^{N_X} \times \mathbb {R} \longmapsto \mathbb {R}^{N_Y}$, that approximates the score function $\nabla \log (p_{\boldsymbol{Y}|\boldsymbol{X}}(\boldsymbol{y}|\boldsymbol{x},t))$ of the time dependent conditional distribution. This is achieved by defining an objective function

6 $$\begin{aligned} \mathscr {L}(\tilde{\boldsymbol{s}})= \frac{1}{2T}\sum _{t=1}^{T} \beta _t \underset{\begin{array}{c} ( \boldsymbol{x}, \boldsymbol{y}_0)\sim p_{\boldsymbol{XY}}\\ \boldsymbol{y}_{t} \sim p(\boldsymbol{y}_{t}|\boldsymbol{y},t) \end{array}}{\mathbb {E}} \left[ \left| \tilde{\boldsymbol{s}}(\boldsymbol{y}_{t},\boldsymbol{x},t) + \frac{\boldsymbol{y}_{t}-\boldsymbol{y}_0}{\beta _t} \right| ^{2}\right] , \end{aligned}$$

where the optimal score network model is given by $\tilde{\boldsymbol{s}}^*= \mathop {\mathrm {arg\,min}}\limits _{\tilde{\boldsymbol{s}}}\, \mathscr {L}(\tilde{\boldsymbol{s}})$. The term within the square parentheses in Equation 6 is similar in form to a regression loss. Therefore, we recognize that the output of a fully trained score network is given by $\tilde{\boldsymbol{s}}^*(\boldsymbol{y}_{t},\boldsymbol{x},t) \approx -({\boldsymbol{y}_{t}-\boldsymbol{y}_0})/{\beta _t}$. By rearranging this equation, we note that $\boldsymbol{y}_0 \approx {\beta _t}\tilde{\boldsymbol{s}}^*(\boldsymbol{y}_{t},\boldsymbol{x},t) + \boldsymbol{y}_{t}$. That is, the value of the score function, when multiplied by the variance $\beta _t$, is what should be added to the perturbed image $\boldsymbol{y}_{t}$ to generate the image without noise ($\boldsymbol{y}_0$) at any step *t*. In practice, the denoising process in NCSN is performed iteratively through *T* steps of Langevin MC^21^, where at any step, a less noisy image $\boldsymbol{y}_{t-1}$ is generated using

7 $$\begin{aligned} \boldsymbol{y}_{t-1} = \boldsymbol{y}_{t} +\gamma _t \tilde{\boldsymbol{s}}^*(\boldsymbol{y}_{t},\boldsymbol{x}, t) + \sqrt{2\gamma _t} \boldsymbol{\epsilon }. \end{aligned}$$

Here $\gamma _t = \lambda _\gamma \beta _t / \beta _{1}$ is the step size, $\lambda _\gamma$ is a hyper-parameter, and $\beta _1$ is the variance for the first time step (typically the smallest $\beta _t$). The sampling process begins by selecting $\boldsymbol{y}_{T} \sim \mathscr {N}(0, \beta _T \mathbb {\boldsymbol{I}})$ and a given input image $\boldsymbol{x}$, and using these in the equation above. This results in $\boldsymbol{y}_{0}$, which is a single sample of the transformed image generated conditioned on $\boldsymbol{x}$. This process is repeated to generate multiple samples.

### Denoising diffusion probabilistic models (DDPM)

The noising process in the DDPM method also involves adding a tailored noise to $\boldsymbol{y}_{0}$,

8 $$\begin{aligned} \boldsymbol{y}_{t}=\sqrt{\bar{\alpha }}_t \boldsymbol{y}_{0}+\sqrt{1- \bar{\alpha }_t}\boldsymbol{\epsilon }, \end{aligned}$$

where $\alpha _t = 1 - \beta _t$ and $\bar{\alpha }_t = \prod _{s=1}^t\alpha _s$^56^ and $\boldsymbol{\epsilon } \sim \mathscr {N}(0, \mathbb {\boldsymbol{I}})$. In the DDPM method, unlike NCSN, the variances are limited to be between zero and one as $\beta _t \in (0, 1), t=1 \dots T$, such that $\beta _1< \beta _2< \dots < \beta _T$. The noisy image $\boldsymbol{y}_t$ generated through Equation 8 and its counterpart from the initial distribution $\boldsymbol{y}_0$ are used to train a denoiser function in the form of a neural network $\tilde{\boldsymbol{\eta }}: \mathbb {R}^{N_Y} \times \mathbb {R}^{N_X} \times \mathbb {R} \longmapsto \mathbb {R}^{N_Y}$. This network is trained using the loss function^56^,

9 $$\begin{aligned} \mathscr {L}(\tilde{\boldsymbol{\eta }})= \frac{1}{2T}\sum _{t=1}^{T} \underset{\begin{array}{c} ( \boldsymbol{x}, \boldsymbol{y}_0)\sim p_{\boldsymbol{XY}}\\ \boldsymbol{\epsilon } \sim \mathscr {N}(0, \mathbb {\boldsymbol{I}}) \end{array}}{\mathbb {E}} \left[ \left| \tilde{\boldsymbol{\eta }}(\sqrt{\bar{\alpha }}_t \boldsymbol{y}_0+\sqrt{1- \bar{\alpha }_t}\boldsymbol{\epsilon },{\boldsymbol{x}},t) - \boldsymbol{\epsilon } \right| ^{2}\right] , \end{aligned}$$

where the optimal denoiser is given by $\tilde{\boldsymbol{\eta }}^*= \mathop {\mathrm {arg\,min}}\limits _{\tilde{\boldsymbol{\eta }}}\, \mathscr {L}(\tilde{\boldsymbol{\eta }})$. Similar to the NCSN, the loss function in Equation 9 resembles a regression loss. Further, by comparing the term within the bracket in Equation 9 and the noising process (Equation 8), we observe that the denoiser learns to estimate a measure of noise added to the clear image; that is, $\tilde{\boldsymbol{\eta }}^*(\boldsymbol{y}_t, \boldsymbol{x},t) \approx \boldsymbol{\epsilon }$.

The DDPM model used in this study^48^ is an extension of an earlier model^56^. The training of this model involves minimization of a hybrid loss that contains an additional term so that the model also learns the sequence of noise scales $\tilde{\beta } (t)$ for $t=1 \dots T$, instead of accepting them as parameters defined by the user. This network is denoted by $\tilde{\beta } (t)$, and it is trained concurrently with $\tilde{\boldsymbol{\eta }}$. The denoising process in the DDPM method is given by

10 $$\begin{aligned} \boldsymbol{y}_{t-1}=\frac{1}{\sqrt{\alpha _t}} \left( \boldsymbol{y}_{t} -\frac{1-\alpha _t}{\sqrt{1-\alpha \bar{}_t}}\tilde{\boldsymbol{\eta }}^*\left( \boldsymbol{y}_t,\boldsymbol{x},t \right) \right) +\sqrt{\tilde{\beta } (t)} \boldsymbol{\epsilon }. \end{aligned}$$

The sampling process begins by sampling $\boldsymbol{y}_{T} \sim \mathscr {N}(0, \mathbb {\boldsymbol{I}})$, selecting an input image $\boldsymbol{x}$, and using these in Equation 10. These iterations end with the $\boldsymbol{y}_0$, which is a sample of the transformed image conditioned on the input image $\boldsymbol{x}$.

The NCSN and DDPM methods share many common features, including beginning with a sample drawn from a simple Gaussian distribution, iteratively denoising this sample using Langevin dynamics, and using a neural network that is trained using a simple regression loss. The key difference between the two methods is in the initial distribution from which samples are generated. In the NCSN method, this is a multivariate Gaussian distribution with zero mean and a very large variance, whereas in the DDPM method, it is a multivariate Gaussian distribution with zero mean but with a variance of unity. The reader is referred to^57^ for a unified description of these methods and their connections with the theory of stochastic differential equations.

### Evaluation of mean and standard deviation images

For all three generative models (cGAN, NCSN, and DDPM), the inference process begins by using a 2D axial image of the brain $\boldsymbol{x}$ as input. Thereafter, in the cGAN model, *n* samples of the latent vectors, $\boldsymbol{z}$ with dimension $N_{Z}=128$ are used as input to the generator, which generates *n* images $\boldsymbol{y}^{(i)}, i = 1, \cdots , n$ from the desired conditional distribution. For the diffusion models, the process is similar; however, the dimension of $\boldsymbol{z}$ is the same as that of $\boldsymbol{y}$ ($128^2$). These samples are treated as the initial condition in the denoising process, which terminates in the generated images $\boldsymbol{y}^{(i)}, i = 1, \cdots , n$. We note that the sampling process in the diffusion models is computationally expensive since it involves $T = 1000$ steps. We also note that we used $n=20$ as the number of samples throughout this work. In Supplementary Notes online, we briefly discuss the impact of this number on the distributional characteristics and accuracy of the outputs.

All three generative models generate an ensemble of images from the conditional distribution, which is used to compute a single pixel-wise mean image, $\bar{\boldsymbol{y}}= \frac{1}{n} \sum _{i=1}^{n} \boldsymbol{y}^{(i)}$ and a pixel-wise standard deviation image $\boldsymbol{y}' = (\frac{1}{n} \sum _{i=1}^{n}{( \boldsymbol{y}^{(i)}-\bar{\boldsymbol{y}})^2})^{{1}/{2}}$, where the operations are interpreted as being pixel-wise.

Transformed images from the DDPM model demonstrate isolated points with hyper or hypo-intense voxel intensities following the process described above. These voxels are identified by looking for outliers in the pixel-wise standard deviation image. They are eliminated by replacing the mean intensity value with its pixel-wise median. We note that the generative capability of the DDPM model allows us to compute the pixel-wise mean, median, and standard deviation values and, therefore, eliminate these outliers (see Supplementary Notes online for a brief note).

### Evaluation metrics and statistical measures

Here, we briefly discuss the metrics used to measure the similarity between individual output images and their counterparts from the test dataset. We also briefly define the statistical measurements and metrics related to binary classification, uncertainty, and distributional behavior of the models discussed throughout this work.

#### Image to image similarity metrics

1) Structural similarity index measure (SSIM) is an image similarity metric that is used in this work to assess the similarity between the outputs of the models and the target images in the form of 3D volumes. SSIM measures the similarity based on local patterns of intensity and incorporates luminance, contrast, and structural information. SSIM values range from zero to one, where a SSIM value of one is indicative of a perfect match. In this work, calculated SSIM values are reported as a percentage. 2) Peak signal-to-noise ratio (PSNR) quantifies the fidelity of a reconstructed image by comparing the maximum signal intensity to the magnitude of the reconstruction error. While higher PSNR typically indicates better image quality, it does not always correlate well with perceived visual similarity^58^. 3) Learned perceptual image patch similarity (LPIPS)^51^ computes the distance between deep feature representations extracted from a pre-trained deep neural network (AlexNet in this work). The LPIPS distance between two arbitrary images $\boldsymbol{y}^{(1)}$ and $\boldsymbol{y}^{(2)}$ is computed as

11 $$\begin{aligned} \text {LPIPS}(\boldsymbol{y}^{(1)}, \boldsymbol{y}^{(2)}) = \sum _{l} \frac{1}{H_l W_l} \sum _{h, w} \left\| w_l \odot \left( \phi _l(\boldsymbol{y}^{(1)})_{h,w} - \phi _l(\boldsymbol{y}^{(2)})_{h,w} \right) \right\| _2^2, \end{aligned}$$

where $\phi _l(\boldsymbol{y}^{(i)})_{h,w}$ is the latent representation of image $\boldsymbol{y}^{(i)}$ at location (*h*, *w*) in layer *l* of the pre-trained neural network. $w_l$ are weights associate to layer *l* for calculating LPIPS, and $\odot$ denotes element-wise multiplication. We note that LPIPS provides a measure of error (not similarity); therefore, lower values of LPIPS indicate better perceptual similarity between $\boldsymbol{x}$ and $\boldsymbol{y}$, with the value of zero indicating a perfect match. 4) The Boundary SSIM (BSSIM) metric is a variant of SSIM that measures similarity between edge maps of images rather than raw intensities of the whole images. By applying SSIM to edges extracted via the Sobel filter, BSSIM emphasizes structural fidelity at region boundaries, highlighting successful tissue reconstruction during transformation. The SCSSIM metric between two arbitrary 3D volumes $\boldsymbol{Y}^{(1)}$ and $\boldsymbol{Y}^{(2)}$ is defined as

12 $$\begin{aligned} \text {SCSSIM} (\boldsymbol{Y}^{(1)}, \boldsymbol{Y}^{(2)}) = 1 - \frac{1}{N-1} \sum _{i=1}^{N-1} \left| \textrm{SSIM}(\boldsymbol{Y}^{(1)}_i, \boldsymbol{Y}^{(1)}_{i+1}) - \textrm{SSIM}(\boldsymbol{Y}^{(2)}_i, \boldsymbol{Y}^{(2)}_{i+1}) \right| , \end{aligned}$$

where $\boldsymbol{Y}^{(1)}_i$ and $\boldsymbol{Y}^{(1)}_i$ denote the *i*-th out of *N* axial slices of the generated and target volumes, respectively. Any sudden changes between consecutive slices, captured by SSIM as a local structural deviation, result in a lower score, reflecting reduced volumetric smoothness. A score of one indicates a perfect match in inter-slice differences between the generated and target images. We also computed the Fréchet Inception Distance (FID) using several pre-trained feature extractors. However, the results did not exhibit consistent trends across models or modalities, in line with prior observations^59^. We speculate that FID quantifies distribution-level similarity and is more suitable for unconditional image generation. Therefore, we focused on LPIPS, which more directly reflects perceptual correspondence between paired MR images.

#### Classification evaluation

1) The F1 score is employed to evaluate the binary classification performance for distinguishing normal inputs from OOD inputs. It is defined as the harmonic mean of precision and recall, providing a single metric that balances false positives and false negatives. In this work, the predicted uncertainty values $\hat{\sigma }$ are converted to binary labels $\hat{y}_i$ using a threshold, and then compared against the true labels $y_i \in \{0,1\}$ (normal or OOD) to determine the counts of true positives (TP), false positives (FP), and false negatives (FN). The F1 score is then computed as

13 $$\begin{aligned} \text {F1} = 2 \cdot \frac{\text {Precision} \cdot \text {Recall}}{\text {Precision} + \text {Recall}}, \text {where} \quad \text {Precision} = \frac{TP}{TP + FP}, \text { and} \quad \text {Recall} = \frac{TP}{TP + FN}. \end{aligned}$$

A higher F1 score indicates better balance between correctly detecting OOD samples and minimizing false alarms, reflecting more reliable classification performance. 2) In binary classification, the Brier score quantifies the mean squared error between the predicted probabilities $\hat{\sigma }_i$ and the true binary labels $y_i \in \{0,1\}$, without converting predictions to discrete labels. It jointly assesses both the accuracy and calibration of probabilistic predictions, where lower Brier scores correspond to better predictive performance. Formally, the Brier score is defined as:

14 $$\begin{aligned} \text {Brier Score} = \frac{1}{N} \sum _{i=1}^N \left( \hat{\sigma }_i - y_i \right) ^2, \end{aligned}$$

where *N* is the total number of samples. 3) In the context of binary classification, the Negative Log-Likelihood (NLL), also known as log loss, is a measure of how well a classifier predicts the true labels. It penalizes confident but incorrect predictions more heavily and is defined as:

15 $$\begin{aligned} \text {NLL} = -\frac{1}{N} \sum _{i=1}^{N} \left[ y_i \log (\hat{\sigma }_i) + (1 - y_i) \log (1 - \hat{\sigma }_i) \right] , \end{aligned}$$

where $y_i$ is the true label and $\hat{\sigma }_i$ is the predicted probability for class OOD. 4) Expected calibration error (ECE) measures the discrepancy between predicted confidence and empirical accuracy. Given *M* confidence bins, ECE is computed as

16 $$\begin{aligned} \text {ECE} = \sum _{m=1}^{M} \frac{n_m}{N} \left| \text {acc}(m) - \text {conf}(m) \right| , \end{aligned}$$

where $n_m$ is the number of samples in bin *m*, and $\text {acc}(m)$ and $\text {conf}(m)$ are the empirical accuracy and average confidence in that bin, respectively. For example, we expect to observe $10\%$ of values within a $10\%$ confidence interval of the distribution of the outputs in a well-calibrated model. ECE provides a measure for calibration error, and a calibration method aims to reduce this error by updating the scores that better represent the true likelihood of outcomes^60^

#### Distributional measures

1) The Shapiro-Wilk test is a statistical normality check that evaluates whether a set of samples is drawn from a normal distribution. In the Shapiro–Wilk test, the null hypothesis is that the data are drawn from a normal distribution. Therefore, for samples that yield a *p*-value greater than the chosen significance level ($p > 0.05$ in this work), we fail to reject the null hypothesis. This means there is no statistically significant evidence to conclude that the samples deviate from normality. 2) Cumulants are statistical descriptors that characterize a probability distribution. They are derived from the cumulant generating function

17 $$\begin{aligned} K(t) = \log \mathbb {E}[e^{tX}]. \end{aligned}$$

The first four cumulants are: the mean $\kappa _1 = \mathbb {E}[X]=\mu$, the variance $\kappa _2 = \text {Var}(X)=\sigma ^2$, the third-order cumulant $\kappa _3 = \mathbb {E}[(X - \mu )^3]$ (skewness), and the fourth-order cumulant $\kappa _4 = \mathbb {E}[(X - \mu )^4] - 3\sigma ^4$. We use the Shapiro-Wilk test and cumulants to analyze the distribution of the outputs in generative models

### Datasets and pre-processing

We use two public datasets with multiple image types, IXI^49^ and OASIS^50^, for training and evaluation of the proposed methods. The IXI dataset consists of 600 MR images of three types: T1-weighted (T1), T2-weighted (T2), and proton density (PD). The scans are collected in three different hospitals in London from normal healthy subjects. Of these, 575 image sets passed QC (those with missing images and realignment issues were removed), from which 357, 74, and 144 images were used for the training, validation, and testing of the models. We balanced our datasets to have the appropriate number of scans from each hospital in the training, validation, and test datasets.

OASIS-3 is a public longitudinal dataset for normal aging and Alzheimer’s disease, consisting of T1, T2, and fluid-attenuated inversion recovery (FLAIR) images. Within this dataset, we used images from the participants that contain all image types scanned in the same MRI session. Out of 243 images, 156, 31, and 56 are used for training, validation, and testing, respectively.

For images in the IXI dataset, we use the FLIRT module of FSL^61^ to realign the 3D T1 images to the spaces of the T2 images, as they have the narrowest axial field of view. This is done by first using T1 images as the reference, as they have the highest resolution, and then using the inverse transformation to provide T1 images in T2 space. For the OASIS dataset, we align T2 and FLAIR images to a reference T1 image. Thereafter, we extract brains from all images by using the T1 image in a brain extraction algorithm^62^ to generate a mask that is applied to all images. Finally, we perform min-max normalization for all images in both datasets.

In addition to the above datasets used for training and evaluating models, we use a set of sample images from three publicly available datasets as out-of-distribution (OOD) inputs. The first set contains seven images with common artifacts from the FSL example box of MR images^63^. This set includes images with a strong RF zipper, two images with motion artifact, significant rotation, reconstruction artifacts, low SNR, and a strong bias field. These are T1 images without any artifact-free or target images from other contrasts available. The second set contains 14 images from patients with multiple sclerosis selected from the MICCAI MS segmentation challenge^64^ (MSSEG). Finally, the last set consists of 14 images with brain tumors taken from the Multimodal Brain Tumor Segmentation Challenge (BraTS) validation dataset^65–67^.

## Conclusions

We have investigated the effectiveness of several deterministic and probabilistic deep learning methods in transforming MR images. To that end, we implemented and evaluated a U-Net-based direct inference and a residual vision transformer model as benchmarks. The former is based on a deterministic formulation, while the latter has a probabilistic formulation but is implemented to generate outputs deterministically. We implemented and extensively evaluated the conditional version of three major generative models, namely, the Conditional Generative Adversarial Network (cGAN), the Denoising Diffusion Probabilistic Model (DDPM), and the Noise Conditional Score Network (NCSN). We trained all these models on the publicly available IXI and OASIS datasets and then applied the trained models to transform T1, T2, FLAIR, and PD MR images. We quantified this performance by computing the SSIM, PSNR, LPIPS, and BSSIM image similarity metrics. We concluded that the two diffusion models consistently yielded more accurate transformations. When comparing two diffusion models, the DDPM model yields higher accuracy, especially with larger samples $(n \ge 5)$ and remains more robust when encountering inputs from slightly different distributions. However, the DDPM model exhibits certain issues. Especially, it produces isolated pixels with outlier values. On the other hand, the NCSN model yields slightly lower accuracy. However, the NCSN model demonstrates several favorable characteristics. For example, it is faster and achieves higher accuracy with a lower number of samples ($n < 5$). Also, the outputs of this model are artifact-free and do not require any additional post-processing.

We also investigated the utility of the generative property of the models, i.e., their ability to produce an ensemble of outputs for a given input image. In particular, in addition to the standard MR images, we incorporated several out-of-distribution (OOD) sets of inputs, including images with artifacts and pathologies, such as Alzheimer’s disease, multiple sclerosis (MS) lesions, and brain tumors. Thereafter, for each input image, we computed the average pixel-wise standard deviation using the three probabilistic models. We concluded that this quantity effectively distinguished OOD images from their regular counterparts in cGAN and NCSN models.

In clinical research, the proposed image transformation models can generate image contrasts that are otherwise unavailable, thereby improving data completeness for downstream analyses. The accompanying uncertainty-based OOD detection provides an automated quality control mechanism that enhances analytical reliability by excluding low-quality data and reducing the time and cost of manual inspection. From a clinical standpoint, this framework could be integrated into image reconstruction or transformation pipelines to automatically flag uncertain outputs for radiologist review. Although deep learning–based models are not yet routinely embedded in clinical workflows, the proposed approach offers a practical and transparent pathway toward uncertainty-aware, quality-controlled image generation.

While the diffusion models (NCSN and DDPM) achieved higher accuracy than the other models, they were also more computationally demanding, particularly during inference. Training times were approximately 42 h for DDPM, 29 h for NCSN, 25 h for cGAN, and 47 h for ResViT (9 h + 38 h for sequential phases). The DDPM and NCSN models required about 15 s and 13 s, respectively, to generate a single slice on an NVIDIA A40 GPU (48 GB), whereas the cGAN, ResViT, and Direct models completed the task in under 0.1 s. Training with a batch size of 16 required approximately at least 16–24 GB of GPU memory, while inference could be performed on less advanced GPUs (e.g., NVIDIA GeForce RTX 2080). The cGAN model required additional memory during training due to its dual-network architecture and the gradient penalty term. Sampling for diffusion models can be further accelerated using batched inputs, resulting in approximately 4 and 2.5 seconds of generation time per image for the DDPM and NCSN models, respectively.

We also conducted several analyses to explore the characteristics of the distribution of the samples produced by the generative models. We used the Shapiro-Wilk test to investigate the normality of the distribution of values generated for individual pixels. We concluded that approximately $89\%$ of distributions in NCSN outputs demonstrate normality. This value is about $51\%$ and $41\%$ for the cGAN and DDPM models, respectively. We further investigated the calibration errors for the outputs of these models. Again, the NCSN model exhibited the best calibration properties among the generative models. In contrast, the cGAN and DDPM model outputs showed overconfidence and underconfidence, respectively.

In this work, we have focused on implementing and quantifying the performance of the proposed models in terms of image similarity metrics and exploring their distributional measures and characteristics for uncertainty quantification. A potential follow-up study could explore the use of transformed images in downstream tasks. For instance, most structural analysis software, like FreeSurfer, primarily rely on T1 MR scans. In the follow-up study, one could consider the use of native T1 images and T1 images obtained from transforming T2 images in FreeSurfer and compare the resulting outputs from the two sets. The outputs would include quantities of clinical interest, such as the volume of brain tissues. An analysis of this type would also provide the groundwork for determining whether MR images of a given type, such as T2 images, can effectively replace T1 images in clinical applications.

## Supplementary Information

Supplementary Information.

## Data Availability

The data generated and/or analyzed during the current study are available from the corresponding author upon reasonable request.
