# Supplementary material for: Probabilistic brain MR image transformation using generative models
Source: Sci Rep. 2025 Dec 20;16:2161. doi: 10.1038/s41598-025-31958-3 (PMC12808115; doi:10.1038/s41598-025-31958-3)
Supplement: Supplementary file 1 — Supplementary Information. [file 41598_2025_31958_MOESM1_ESM.pdf]

Supplementary Notes

Impact of number of samples (ensemble size n)

The number of generated samples for all generative models is set to be $n = 20$ in this work. Figure S1 demonstrates how varying n affects the outputs of the generative models. In this figure, the first two plots (from left to right) show the averaged SSIM and PSNR for the T1-to-T2 transformation on the IXI dataset as a function of the number of generated samples. As shown, both SSIM and PSNR exhibit improvements by increasing the number of samples and then plateau after a certain point. Similar behavior has been reported in prior studies (e.g.,[1]). Among the models, DDPM shows the least stability and higher gains by increasing the number of samples. The rightmost plot demonstrates the gain in SSIM, the primary accuracy metric, with respect to an estimate of additional computational cost (generation time for a single image, optimized through batch generation). Consistent with previous discussions, the NCSN network achieves minimal improvements (less than 0.5%) beyond $n = 5$ ($t \approx 12.5$ s), whereas the DDPM model continues to gain accuracy with additional computational time (up to $n = 8$, at the cost of $t \approx 32$ s).

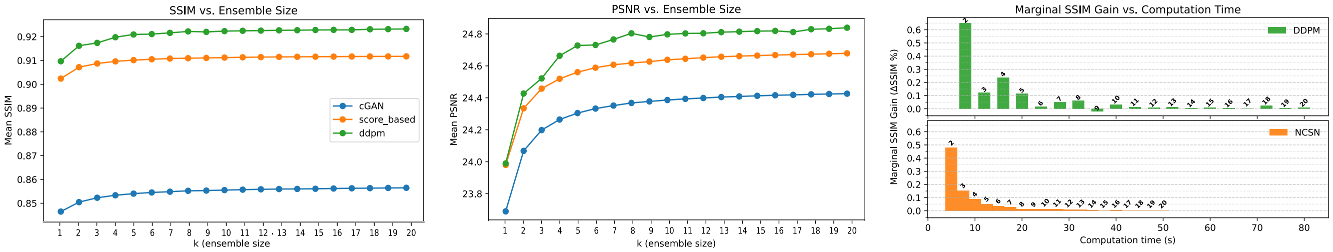

Supplementary Figure S1. From left to right, the impact of the number of samples (n) on the calculated average for SSIM and PSNR for cGAN, NCSN, and DDPM models. The rightmost plot shows the gain in SSIM accuracy (through additional samples) based on an estimated time required to generate an image. This plot aims to provide a practical guide for selecting the number of samples n under computational budget constraints.

To illustrate the impact of the number of samples on the output of the generative models, we also reproduce Table 4 of the manuscript with $n = 1$ in Table S1 for cGAN, NCSN, and DDPM models. Additionally, we show the changes from $n = 20$ samples to a single sample in parentheses. For SSIM, PSNR, and BSSIM, an increase in values (positive sign) indicates improvement in performance, while for LPIPS, a decrease in values (negative sign) indicates improvement, and vice versa. As can be seen, the NCSN model shows the best performance and lowest performance drop when working with a single sample ($n = 1$). The DDPM model shows greater performance loss with a single output (or, equivalently, greater performance gain with a higher number of samples). This observation aligns with the discussions presented for Figure S1 and can be attributed to the broader range of values generated in DDPM outputs, as also shown in Figure 9 of the manuscript.

Supplementary Table S1. Average similarity metrics (SSIM, PSNR, LPIPS, and BSSIM) for the cGAN, NCSN, and DDPM models using the number of samples equal to one ($n = 1$). The values in parentheses indicate the changes in performance compared to outputs generated from $n = 20$ samples.

		cGAN	NCSN	DDPM
IXI	SSIM	89.62 (-3.04)	92.55 (-1.81)	**93.52** (-1.88)
	PSNR	21.38 (-2.32)	**24.88** (-0.55)	23.84 (-5.89)
	LPIPS	0.056 (+0.001)	0.037 (-0.007)	**0.036** (+0.013)
	BSSIM	87.18 (-1.23)	90.98 (-0.37)	**91.70** (-2.38)
OASIS	SSIM	87.95 (-1.48)	**91.10** (-1.11)	89.14 (-2.98)
	PSNR	20.98 (-0.42)	23.20 (+0.66)	**23.84** (-1.30)
	LPIPS	0.067 (+0.005)	**0.042** (-0.005)	0.058 (+0.017)
	BSSIM	85.29 (-1.58)	**89.30** (-1.19)	86.01 (-3.64)
COMBINED	SSIM	89.15 (-2.61)	**92.14** (-1.62)	**92.29 (-2.19)**
	PSNR	21.27 (-1.79)	24.41 (-0.21)	**26.25** (-2.19)
	LPIPS	0.059 (0.002)	**0.039** (-0.006)	0.042 (+0.013)
	BSSIM	86.65 (-1.33)	**90.50** (-0.61)	90.11 (-2.73)

We also show the impact of n on the cumulants of the distribution of the outputs for the T1-to-T2 transformation on the IXI dataset in Figure S2 (See Evaluation metrics and statistical measures for definitions). We observe that while the overall average

(k_1) is stable for lower values of n, the average standard deviation (k_2) continues to increase until around $n = 15$, suggesting a need for sufficient samples for uncertainty estimation. These results suggest that, depending on the application, generating more than 20 samples is unlikely to yield substantial benefits in terms of either output quality or uncertainty representation. We observe similar behavior for discussions presented in Figure 9 of the manuscript, S1, and S2 for transformations other than T1-to-T2 on the IXI dataset.

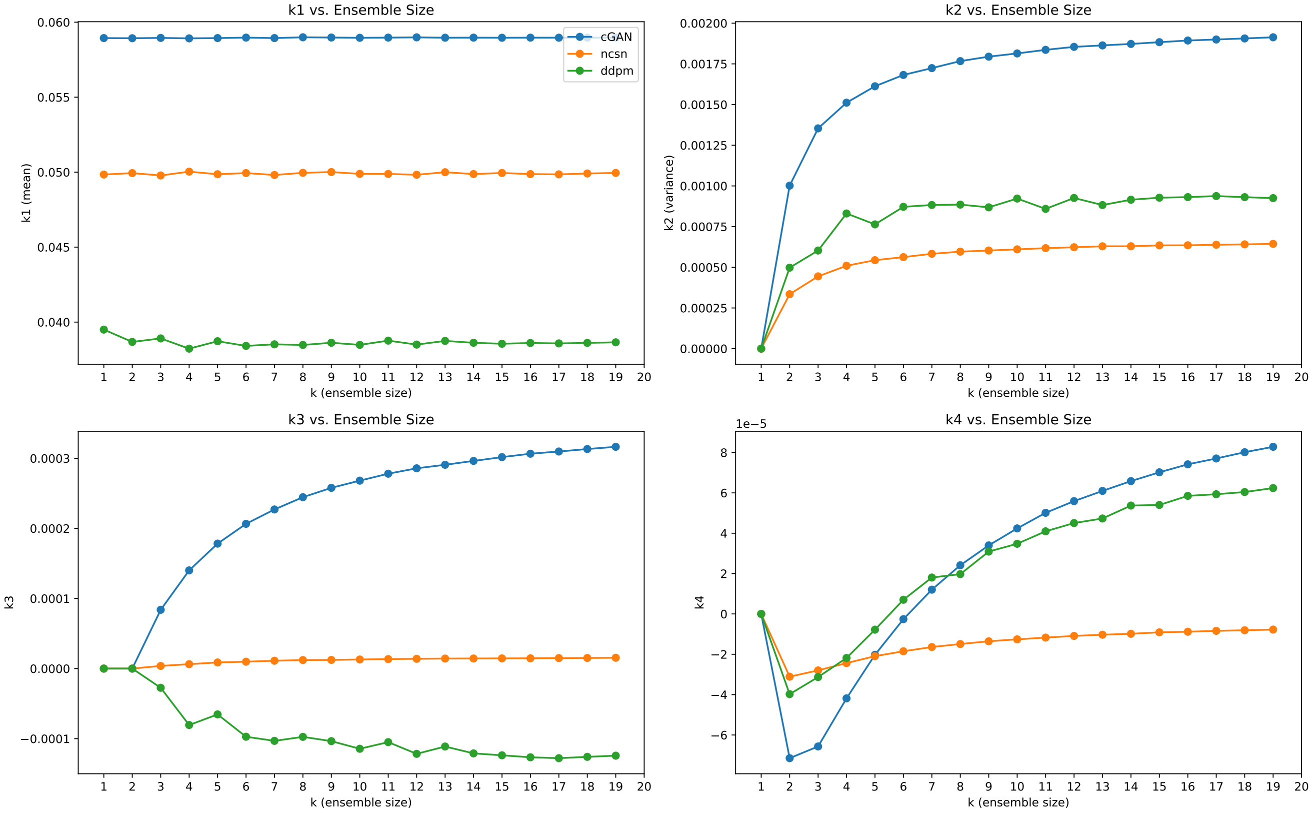

Supplementary Figure S2. The impact of the number of samples (n) on the calculated cumulants (k_1 to k_4) for cGAN, NCSN, and DDPM models.

In an additional analysis, Table S2 presents the results as the percentage of pixel distributions that pass the Shapiro–Wilk test for each model. We observe from Table S2 that the NCSN model outputs demonstrate significantly higher normality compared to the other two models. In contrast, the cGAN results are the worst in terms of normality based on the Shapiro–Wilk test. Our further measurements (not reported here for brevity) reveal that the NCSN model yields skewness values close to zero, while the DDPM and cGAN models show negative and positive skewness values, respectively. We also observed large positive kurtosis values for the cGAN and DDPM models, and values closer to zero for the NCSN model. All these values suggest that the pixel values in the output of the NCSN model are more likely to follow a univariate normal distribution.

Supplementary Table S2. The percentage of pixel value distributions in the generated samples that pass the Shapiro-Wilk normality test.

	cGAN	NCSN	DDPM
IXI	46.85	**89.15**	51.17
OASIS	26.86	**89.37**	51.04
COMBINED	41.25	**89.21**	51.13

Notes on network architectures and hyperparameters

We aimed to keep the hyper-parameters consistent across the models whenever possible while adhering to the original implementation of each work. Accordingly, we used the same training and testing data for all models with an image resolution of 128×128 and a batch size of 16. The learning rate is set to $1e-4$ with Adam as the optimizer and amsgrad[2] option enabled

in all training experiments. The neural network architecture details for the cGAN critic and generator with conditional instance normalization (CIN)[3] can be found in[4] and[5]. We replaced the ResBlock of the generator U-Net with a Dense block discussed in[6]. Also, the details of the U-Net for the DDPM can be found in[1] and[7], and for the NCSN models in[8] and[9]. We note that we adopted the U-Net architecture used in the DDPM model for the Direct model by eliminating the time embedding layers. The number of channels for the first level in the U-Nets (network growth factor) is chosen to be 64 for all models, resulting in 20 ± 2 million trainable parameters (weights) for all models, except for the cGAN critic, which has 5.8 million weights. For both diffusion models (DDPM and NCSN), we used 1000 diffusion steps. As previously mentioned, σ_t are learned during the training by the model in the DDPM method. For NCSN, the authors propose a technique to determine the initial noise scale. They suggest setting the largest noise scale to be as large as the maximum pairwise Euclidean distance of training data points. We note that this value differs depending on the dataset and should be calculated based on the output image type dataset (as the noising process is applied to the output image). Also, using the maximum Euclidean norm of all data points can be a reasonable estimate for the maximum noise scale in MR images. For all models, we used 0.01 as the minimum noise scale.

For the ResViT model, we closely followed the instructions provided in the original repository of the paper[10]. This involved using pre-trained ViT models, pre-training the ART blocks without the transformer components, and then performing a final fine-tuning phase. All hyperparameters were kept as suggested. However, instead of loading training data from .png files as in the default setup, we used NumPy arrays directly, similar to other models. We also retained the patch size of 16×16 but configured the transformer grid to 8×8 to match the input image resolution.

As we mentioned in the manuscript, we observed isolated spots in the output of the DDPM model with hypo or hyper-intense out-of-range values. We performed a quantitative analysis of outlier voxels across generated samples and found that the outliers are rare (13.8 per million voxels). However, given that a slice contains 128×128 pixels, this implies that they appear in about 22.6% of slices. We also observed that across the multiple samples generated for a given input, they seldom occur at the same voxel. Therefore, a median filter, where the median is computed for the pixel in question across $n = 20$ samples, effectively removes them without introducing noticeable distortions. Here, we also provide some additional notes. We observed that large gradients occur during the training of the DDPM model for this particular problem. Incorporating gradient clipping and normalization and enabling amsgrad[2] in the Adam optimizer with lower than usual coefficient values ($\beta_1 = 0.2$ and $\beta_2 = 0.7$) mitigated this problem but could not fully resolve it. We also experimented with the same neural network architecture in other frameworks and the DDPM model in other problems (not reported here) and did not face this issue. Therefore, we speculate that utilizing DDPM for medical image transformation requires more carefulness.

Supplementary References

1. Wolleb, J., Sandkühler, R., Bieder, F., Valmaggia, P. & Cattin, P. C. Diffusion models for implicit image segmentation ensembles. In *International Conference on Medical Imaging with Deep Learning*, 1336–1348 (PMLR, 2022).

2. Reddi, S. J., Kale, S. & Kumar, S. On the convergence of adam and beyond. *arXiv preprint arXiv:1904.09237* (2019).

3. Dumoulin, V., Shlens, J. & Kudlur, M. A learned representation for artistic style. *arXiv preprint arXiv:1610.07629* (2016).

4. Adler, J. & Öktem, O. Deep bayesian inversion. *arXiv preprint arXiv:1811.05910* (2018).

5. Moazami, S., Ray, D., Pelletier, D. & Oberai, A. A. Probabilistic brain extraction in mr images via conditional generative adversarial networks. *IEEE Transactions on Med. Imaging* (2023).

6. Guan, S., Khan, A. A., Sikdar, S. & Chitnis, P. V. Fully dense unet for 2-d sparse photoacoustic tomography artifact removal. *IEEE journal biomedical health informatics* **24**, 568–576 (2019).

7. Nichol, A. Q. & Dhariwal, P. Improved denoising diffusion probabilistic models. In *International Conference on Machine Learning*, 8162–8171 (PMLR, 2021).

8. Song, Y. & Ermon, S. Improved techniques for training score-based generative models. *Adv. neural information processing systems* **33**, 12438–12448 (2020).

9. Batzolis, G., Stanczuk, J., Schönlieb, C.-B. & Etmann, C. Conditional image generation with score-based diffusion models. *arXiv preprint arXiv:2111.13606* (2021).

10. Dalmaz, O., Yurt, M. & Çukur, T. Resvit: residual vision transformers for multimodal medical image synthesis. *IEEE Transactions on Med. Imaging* **41**, 2598–2614 (2022).
